# Personalized cancer vaccine design using AI-powered technologies

**DOI:** 10.3389/fimmu.2024.1357217

**Published:** 2024-11-08

**Authors:** Anant Kumar, Shriniket Dixit, Kathiravan Srinivasan, Dinakaran M, P. M. Durai Raj Vincent

**Affiliations:** ^1^ School of Bioscience and Technology, Vellore Institute of Technology, Vellore, India; ^2^ School of Computer Science and Engineering, Vellore Institute of Technology, Vellore, India; ^3^ School of Computer Science and Engineering, Vellore Institute of Technology, Chennai, India; ^4^ School of Computer Science Engineering and Information Systems, Vellore Institute of Technology, Vellore, India

**Keywords:** cancer vaccine, artificial intelligence, epitope design, neoantigen prediction, MHCpeptide binding prediction, nucleic acid cancer vaccines, peptide cancer vaccines, personalized cancer vaccine

## Abstract

Immunotherapy has ushered in a new era of cancer treatment, yet cancer remains a leading cause of global mortality. Among various therapeutic strategies, cancer vaccines have shown promise by activating the immune system to specifically target cancer cells. While current cancer vaccines are primarily prophylactic, advancements in targeting tumor-associated antigens (TAAs) and neoantigens have paved the way for therapeutic vaccines. The integration of artificial intelligence (AI) into cancer vaccine development is revolutionizing the field by enhancing various aspect of design and delivery. This review explores how AI facilitates precise epitope design, optimizes mRNA and DNA vaccine instructions, and enables personalized vaccine strategies by predicting patient responses. By utilizing AI technologies, researchers can navigate complex biological datasets and uncover novel therapeutic targets, thereby improving the precision and efficacy of cancer vaccines. Despite the promise of AI-powered cancer vaccines, significant challenges remain, such as tumor heterogeneity and genetic variability, which can limit the effectiveness of neoantigen prediction. Moreover, ethical and regulatory concerns surrounding data privacy and algorithmic bias must be addressed to ensure responsible AI deployment. The future of cancer vaccine development lies in the seamless integration of AI to create personalized immunotherapies that offer targeted and effective cancer treatments. This review underscores the importance of interdisciplinary collaboration and innovation in overcoming these challenges and advancing cancer vaccine development.

## 1 Introduction

Cancer is a major global cause of death (1), responsible for around 10 million deaths in 2020 (2). As one of the leading health challenges worldwide, it remains a critical area of research and innovation. Among the various treatment approaches, immunotherapy has gained significant attention due to its ability to harness the body's own immune system to fight cancer (2). One promising immunotherapy strategy involves the development of cancer vaccines, which are designed to stimulate the immune system to recognize and attack tumor cells. These vaccines work by targeting tumor antigens (TAs), which are unique proteins or molecules expressed on cancer cells. By artificially inducing an immune response against these antigens, cancer vaccines aim to generate specific and long-lasting immunity against the tumor (3). Unlike conventional therapies, cancer vaccines offer the potential for a safer, more targeted, and better-tolerated approach (4). However, their clinical translation has faced numerous challenges, primarily due to the heterogeneous landscape of tumor antigens and the inherent variability of individual immune responses (5, 6).

Although several cancer vaccines, such as Cervarix (7), Gardasil (8), Gardasil-9 (9), and the Hepatitis B vaccine (HEPLISAV-B) (10), have been approved primarily for preventive purposes, advanced vaccines under research aim to specifically target markers like TAAs or neoantigens on cancer cells to activate an immune response and effectively attack these tumors (10). AI has emerged as a transformative tool in this area, significantly accelerating the development of innovative cancer vaccines (11, 12). The availability of vast amounts of public data has further bolstered these advancements, providing researchers with unprecedented opportunities to identify and validate novel targets. This review explores the dynamic interplay between AI and cancer vaccine development, offering insights into how AI can revolutionize this field by enhancing the speed and precision of vaccine discovery and optimization. Recent strides in vaccine technology and coupled with a deeper understanding of cancer immunology have paved the way for innovative therapeutic avenues. AI’s intervention in the cancer landscape has been profound and multifaceted, with significant implications for treatment and research (13). Through data-driven pattern recognition, AI has proven instrumental in detecting mutations and unraveling intricate genomic signatures (14). The integration of modern immunology and data science has introduced innovative analytical methods for vaccine production. Deep learning models, for instance, are capable of exploring a wide range of possibilities in basic, translational, and clinical research, accelerating the development of highly effective cancer vaccines. Moreover, AI can classify patients as responders or non-responders to cancer vaccines, enabling more personalized treatment strategies, improving patient outcomes, and offering alternative therapies for those who do not respond to conventional vaccines.

Pioneering platforms like IBM's Watson Oncology have demonstrated AI's potential in personalized cancer treatment by utilizing vast data repositories to provide tailored therapeutic recommendations (15). Similarly, AI is making significant progress in the field of immunology, particularly in epitope prediction—a crucial step in vaccine development. One notable tool in this domain is DiscoTope-2.0 (12), which calculates the epitope propensity of each residue by analyzing local geometry, including side chain orientation and solvent accessibility. This tool has been instrumental in predicting B-cell epitopes, which are critical for designing effective vaccines. Building on this foundation, DiscoTope-3.0 (16) introduces a more sophisticated approach by integrating a positive-unlabeled learning technique and innovative inverse folding structure representations. Unlike its predecessor, DiscoTope-3.0 is versatile, applicable to both predicted and experimentally solved structures. Most importantly, DiscoTope-3.0 (16) overcomes the limitations of previous models by maintaining high predictive accuracy even for relaxed and anticipated structures, thereby eliminating the dependency on experimental structural data. This advancement not only accelerates the epitope mapping process but also expands the range of antigens that can be analyzed, making it a powerful tool for vaccine research and development.

The road to effective cancer vaccine design is fraught with challenges, from identifying optimal targets to overcoming preexisting immunological tolerance. The complexity deepens when considering mutated antigens, patient-specific variations, and the dynamic evolution of tumor antigens. As AI continues its ascendant trajectory, this review aims to demonstrate the synergy between AI and cancer vaccine design processes. It takes complex biological concepts and makes them easier to understand for both biologists and computer experts. Ultimately, this review ushers in a new era in cancer vaccine development, where AI and human ingenuity coalesce to combat one of the most pressing health challenges of our time. While AI and ML (Machine Learning) technologies can expedite certain aspects of vaccine development, they cannot replace the essential validation provided by animal models and human trials in ensuring safety and efficacy (17). However, this review does not cover all aspects of cancer vaccine research. It specifically excludes topics such as regulatory challenges, large-scale clinical trial design, and the detailed immunological mechanisms involved in T-cell activation and immune memory formation, which are beyond the scope of AI-driven approaches. The ethical implications of AI use in healthcare are discussed briefly, but they are not the primary focus of this review.

## 2 Review methodology

This thoughtful arrangement facilitates exploration of this field for both novice and experienced researchers. To our knowledge, no previous research has thoroughly examined these techniques, specifically the synergistic combination of AI-related technologies with the latest advancements in vaccine technology. We have also identified persisting challenges associated with AI-based tools and proposed innovative research directions to address them. Such combined efforts can facilitate the refinement and widespread adoption of advanced AI technologies for developing effective vaccine-based cancer therapies. The Appendix section includes **Table A1**, which provides a glossary of abbreviations used throughout the article.

### 3.1 Search strategy and literature sources

**Figure 1:**
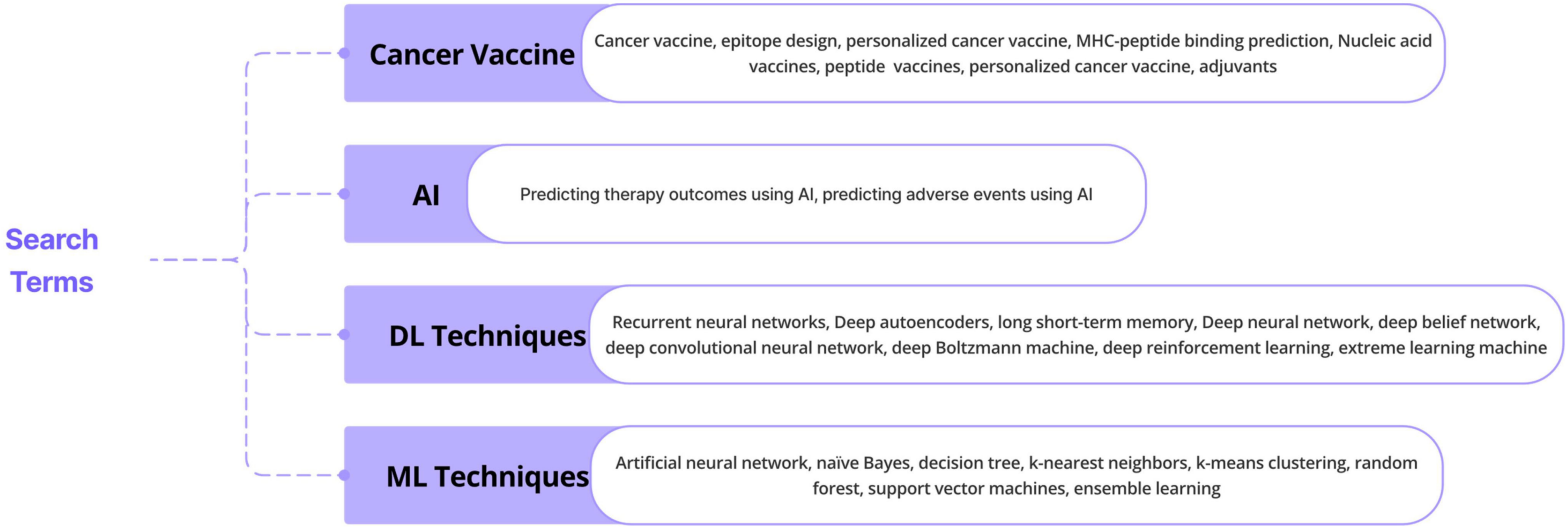
Queries made using specific keywords in databases.

The database queries made with particular keywords are shown in **Figure 1**. This review, adhering to a rigorous literature review approach, presents key findings from research and review papers exploring the application of ML and DL techniques in cancer-based vaccine therapy. Employing the PRISMA-ScR methodology, articles were carefully selected from reputable online databases including Google Scholar, Medline, PubMed Central, Cochrane Library, and Scopus. The literature extensively documents the utilization of these tools in diverse areas like epitope design, mutation prediction, and AI integration in various nucleic acid vaccine designs. This growing body of evidence highlights the expanding recognition of AI technologies in healthcare, offering valuable insights and significant advancements in crucial areas of practice and research. This review further analyzes the contributions of other scholars and proposes insightful directions for future research endeavors.

### 3.2 Inclusion criteria

The article selection process was restricted to English-language publications, prioritizing the novelty and relevance of the review topic. Specifically concentrating on vaccines, we meticulously curated comprehensive articles utilizing ML and deep learning (DL) methodologies in cancer therapy. Only peer-reviewed papers were considered, with an additional requirement of providing original data or analyses.

### 3.3 Elimination criteria

The exclusion process commenced with screening abstracts, followed by data extraction and comprehensive analysis of full texts. Articles were excluded based on multiple criteria, including subpar writing quality, non-English language, or lack of relevance. Duplicate publications and those unrelated to the research topic were also eliminated from consideration. We also didn’t include papers from those journals that are considered predatory.

### 3.4 Results

**Figure 2:**
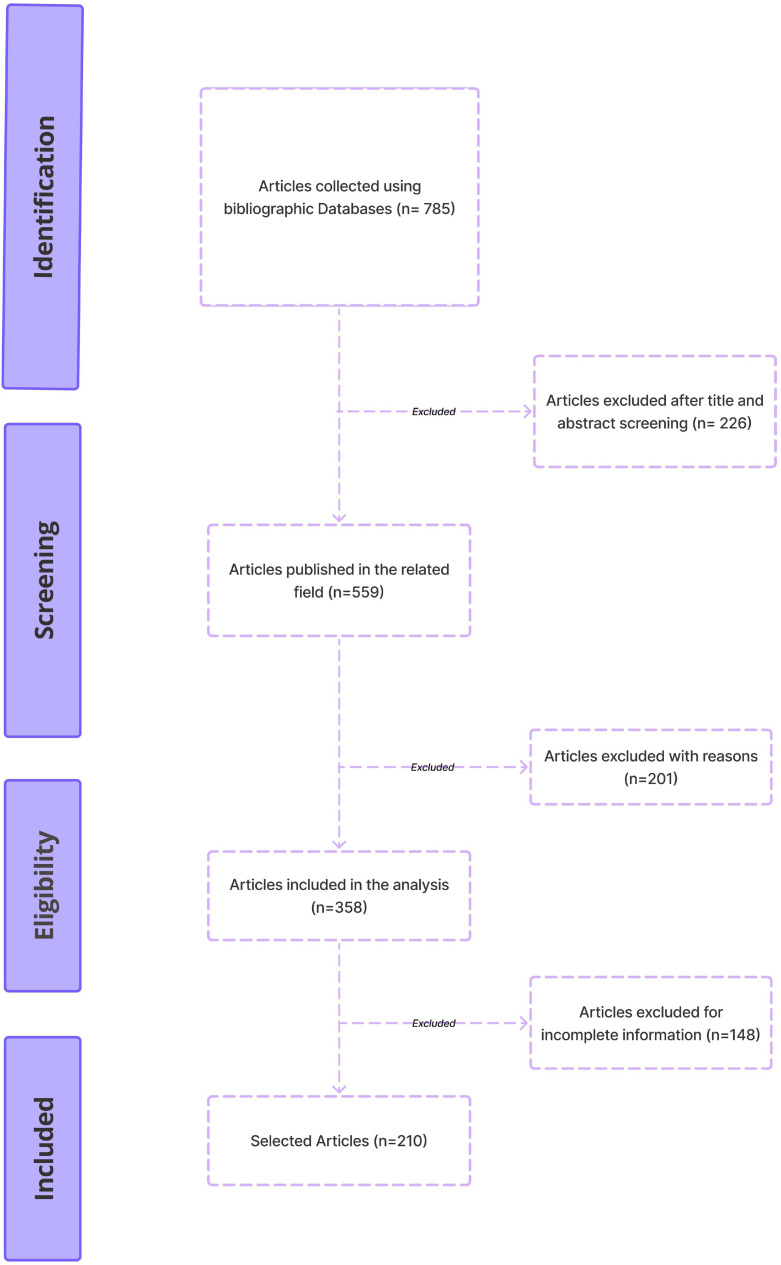
The PRISMA-ScR guidelines-based methodology for selecting studies to be included in the review.

785 publications were obtained from various literary sources, Scopus, Medline, PubMed Central, Cochrane Library, and Google Scholar. 226 Papers were eliminated after titles and abstracts were screened. After thoroughly reviewing the full text of the remaining 358 publications, we further narrowed down the selection. This resulted in a final set of 210 papers chosen for further analysis. The outcomes of this selection process are illustrated in **Figure 2**.

## 4 Conventional vaccine design process

**Figure 3:**
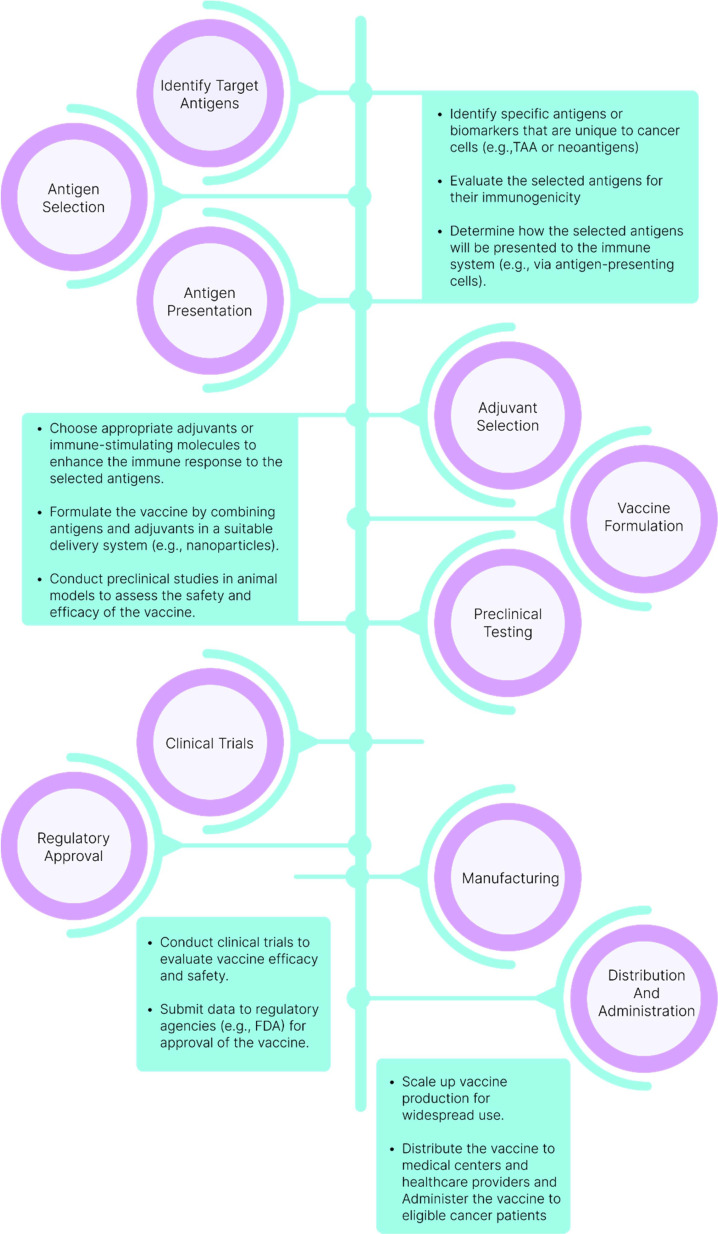
Traditional Vaccine Design Process. The journey of vaccine development commences with the identification and selection of target antigens, which are then combined with suitable adjuvants. Preclinical testing is conducted before progressing to clinical trials. Following the assessment of the vaccine’s efficacy and safety in clinical trials, regulatory approval is sought for large-scale manufacturing and distribution.

Therapeutic cancer vaccines (TCVs) aim to control tumor growth, eliminate residual disease, and induce regression of established tumors. The traditional process of vaccine design, as illustrated in **Figure 3**, highlights the critical steps involved in creating an effective cancer vaccine. Central to this process is the efficient delivery of antigens to dendritic cells (DCs), which leads to their activation and subsequently triggers robust immune responses, including CD4+ T-helper cells and cytotoxic T lymphocytes (CTLs) (18).

**Figure 4:**
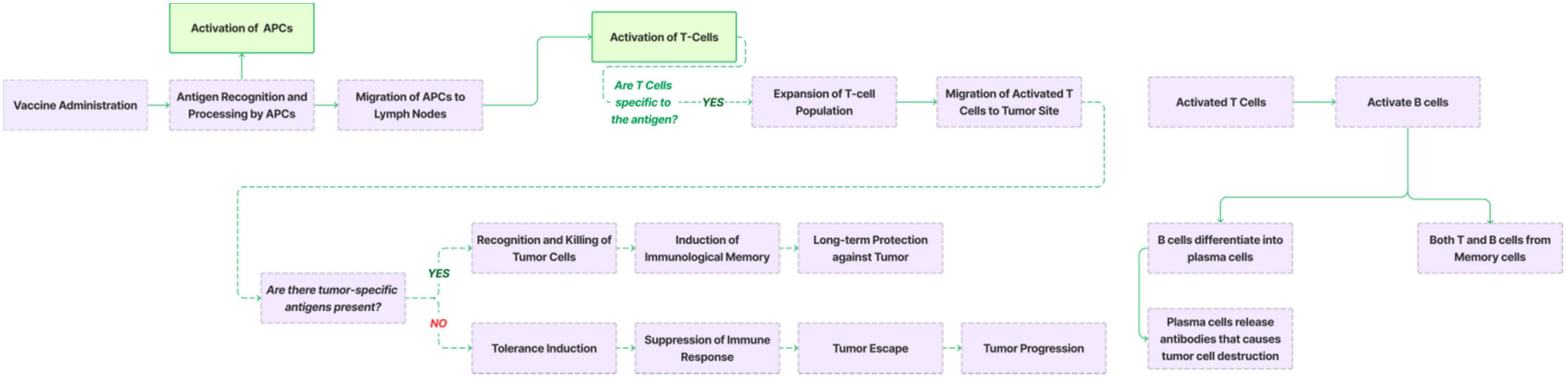
Mechanism of action (MOA) of a vaccine. Cancer vaccines introduce tumor-associated antigens or tumor-specific antigens to the immune system. Antigen-presenting cells (APCs), such as dendritic cells, process these antigens and present them in a Human Leukocyte Antigen (HLA)–restricted manner to T cells. Activated T cells recognize and bind to tumor cells expressing the same antigens, leading to the activation of cytotoxic T cells (CD8+ T cells) and helper T cells (CD4+ T cells). CD8+ T cells directly target and kill tumor cells, while CD4+ T cells assist other immune responses. Activated B cells produce antibodies that neutralize tumor cells or their secreted factors, contributing to tumor cell death. Immune surveillance by T cells and B cells monitors for and eliminates any remaining tumor cells that may have escaped initial treatment. The immune system retains the memory of tumor antigens, allowing a rapid response if the tumor recurs. Additionally, cancer vaccines are often combined with other immunotherapies or standard treatments to enhance efficacy.

Many vaccines are specifically formulated with pathogen-associated molecular patterns (PAMPs), components of pathogens that are recognized by pattern recognition receptors (PRRs) on the surface of dendritic cells. This interaction is key to initiating the immune response. The mechanism of action (MOA) of TCVs is further illustrated in **Figure 4**, where the binding of PAMPs to PRRs sparks an intracellular signaling cascade. This cascade activates DCs by inducing cytokine release and elevating the expression of co-stimulatory molecules on their surface (19).

Once activated, dendritic cells undergo a maturation process that enhances their ability to present antigens to T-cells. This maturation, detailed in **Figure 4**, involves changes in surface molecules, notably the increased expression of MHC molecules and co-stimulatory molecules like CD80 and CD86. The DCs then process the antigens into peptide fragments, which are presented on MHC molecules on their surface.

Following maturation, the DCs migrate from the site of antigen encounter (such as the vaccination site) to nearby lymph nodes. In these lymph nodes, the DCs interact with naive T-cells, presenting antigen-MHC complexes along with co-stimulatory signals, which leads to the activation of the T-cells. As depicted in **Figure 4**, CD4+ T-cells differentiate into helper T-cells, while CD8+ T-cells become cytotoxic T-cells (20). Helper T-cells stimulate B cells to produce antibodies, while cytotoxic T-cells directly attack and eliminate infected or cancerous cells.

The method of vaccine administration plays a crucial role in its effectiveness and safety. While intramuscular injection remains the most commonly used route and is considered the gold standard for inducing systemic immunity, it has limitations in providing mucosal protection. Intradermal and intranasal methods are also preferred for certain vaccines, as they allow direct access to dendritic cells (DCs) in the skin or mucosal tissues, enhancing immune response at these critical sites. Intradermal injection, though requiring a specialized technique, is efficient and allows for the use of smaller doses of the vaccine. Intranasal delivery directly targets mucosal surfaces, promoting both local and systemic immune responses, but often requires the addition of adjuvants to enhance efficacy. Subcutaneous injection offers ease of administration, though it can be less efficient in stimulating a strong immune response. Oral vaccines, while convenient, face challenges such as degradation in the digestive system and reduced immunogenicity. Understanding the advantages and limitations of each administration route is crucial for optimizing vaccine design and delivery strategies. Selecting the most appropriate method can significantly impact the vaccine’s ability to combat various pathogens effectively and ensure robust protection for individuals (21).

Several strategies can be employed to enhance T-cell responses in vaccines, as a more robust and durable immune response is essential for effective cancer immunotherapy. One key approach involves the use of adjuvants, substances that boost the immunogenicity of antigens by stimulating antigen-presenting cells (APCs), such as dendritic cells (DCs), and promoting cytokine production. In addition to adjuvants, targeting specific dendritic cell receptors represents a promising novel strategy. This method focuses on the localized delivery of antigens through antibodies specifically designed to bind to endocytic receptors expressed on the surface of DCs (22). Among the most promising targets for DC-based vaccination is DEC-205, a DC-specific endocytic receptor that efficiently internalizes antigens and directs them to the MHC class II pathway (23). Preclinical models have demonstrated that targeting DEC-205 with antigen-specific antibodies elicits both robust and long-lasting humoral and cellular immune responses. Additionally, other DC-specific receptors, such as Clec9A (24) and Clec12A (25), have shown potential as targets for enhancing DC-based vaccines. By selectively targeting these receptors, vaccines can activate the immune system more effectively, leading to stronger T-cell responses.

Moreover, tailoring the immune response by targeting specific DC subsets can further refine the outcome, steering the immune system towards a more desirable and effective response. An essential consideration is the tumor microenvironment (TME), where sustained infiltration of immune cells and long-term maintenance of the immune response are critical for successful tumor control. One strategy involves utilizing autologous DCs loaded with tumor antigens derived from dying tumor cells within the TME (26, 27). However, these approaches are met with several challenges, including tumor heterogeneity, where different regions of the tumor may express different antigens, potentially leading to an incomplete immune response. Furthermore, insufficient presentation of complex antigens may result in suboptimal activation of T-cells, limiting the overall therapeutic effect. There is also the risk that the immune system might not mount a sufficiently robust attack against tumor cells, as it could perceive tumor antigens as self-antigens, reducing the immune response. Therefore, the design and development of cancer vaccines are intricate and multi-step processes that require careful consideration. This involves identifying specific cancer antigens, evaluating their immunogenicity, formulating the vaccine with appropriate adjuvants, and conducting thorough preclinical and clinical testing. The process progresses through various phases of safety and efficacy evaluation, ultimately leading to regulatory approval and large-scale manufacturing.

## 5 AI in various steps of vaccine development

AI can assist in feature extraction and model training to predict patient-specific cancer antigens. Through sophisticated algorithms, AI may optimize and refine these antigens, guide vaccine formulation, and support clinical trial design, potentially enabling more personalized vaccination strategies. Real-time monitoring and continuous learning could ensure adaptive treatment strategies, while adherence to regulatory standards and experimental validation might help assess safety and efficacy. As AI-driven cancer vaccine development continues to advance, ensuring the safety, efficacy, and ethical use of these technologies requires careful navigation of a complex regulatory landscape. Key challenges may include guaranteeing that AI algorithms do not introduce unintended risks or biases, maintaining transparency throughout development, and addressing potential biases in the training data. Robust data security and privacy measures, along with well-designed clinical trials, will likely be critical for achieving regulatory approval and maintaining public trust. Furthermore, collaboration with regulators and stakeholders globally could help establish harmonized standards, ensuring that ethical considerations like informed consent are properly addressed. This collaboration might ultimately pave the way for the safe and responsible integration of AI-driven vaccines into cancer treatment. 

The development of AI models for cancer vaccine design relies heavily on access to extensive and high-quality datasets. **Table 1** consolidates various datasets and databases that could aid in the creation of novel cancer vaccines. Many of the listed databases are epitope databases, such as IEDB and SYFPEITHI, as well as neoantigen peptide databases like dbPepNeo2, and MHC binders like MHCBN. These resources can provide crucial information on cancer vaccine target antigens and the MHC molecules capable of binding to them, supporting researchers in identifying promising vaccine candidates.

**Table 1:** List of several cancer vaccine datasets.

Ref.	Database Name	Year	Availability	Details about the dataset	Link	Significance
Schuler MM et al. (28)	SYFPEITHI	2007 (last updated - 2012)	Publicly available	A collection of 7000 peptide sequences capable of binding to both class I and class II MHC molecules	http://www.syfpeithi.de/	Therapeutic cancer vaccine based on peptides
Schisler NJ et al. (29)	IEDB	2000 (Last Updated: September 10, 2023)	Publicly available	1,597,734 Peptidic Epitopes and 3,187 Non-Peptidic Epitopes.	https://www.iedb.org/	Therapeutic cancer vaccine based on peptides
Lu M et al. (30)	dbPepNeo2.0	2022	Publicly available	801 high-confidence neoantigens and 864,884 low- confidence peptidomes, validated neoantigen peptides, TCRs, and HLA peptidomes	http://www.biostatistics.online/dbPepNeo2	Neoantigens, which may serve as targets for immunotherapy, can be predicted and filtered using it.
Lata S et al. (31)	MHCBN	2009	Publicly available	MHCBN contains information on 20,717 MHC binders, 4022 MHC non-binders, 1053 TAP binders and non-binders, and 6722 T cell epitopes.	https://webs.iiitd.edu.in/raghava/mhcbn/	Utilizing this dataset enables the prediction of MHC class I binding peptides, a crucial aspect in the development of vaccines.
Kardani K et al. (32)	CPPsite 2.0	2021	Publicly available	Contains over 1000 new entries and includes information on diverse chemical modifications of CPPs.	http://crdd.osdd.net/raghava/cppsite/	CPPsite 2.0 represents an enhanced iteration of the manually curated database dedicated to cell-penetrating peptides (CPPs).
Almeida LG et al. (33)	CTdatabase	2008	Publicly available	Contains information about family members, genomic spots, splicing variations, and gene names and aliases.	http://www.cta.lncc.br	This has data about cancer-testis antigens that can be used as potential targets.
Fang LT et al. (34)	Somaticseq	2015	Publicly available	Contains tumor-normal pairs that were produced using BAMSurgeon with synthetic but accurate genetic mutations.	https://dreamchallenges.org/	The dataset is excellent for evaluating the effectiveness of bioinformatics algorithms in detecting somatic mutations because it covers many stages with increasing difficulty, such as multiple sub-clonal groups and simulated contamination.
Bulik et al. (35)	–	2018	Publicly available	HLA–MS neoantigen peptides and genomic data of 74 patients.	http://massive.ucsd.edu/	The provided dataset can be used to locate new antigens in various cancer types.

### 5.1 Epitope design and major histocompatibility complex binding prediction using AI

An epitope is a specific molecular structure or region on an antigen that the immune system recognizes and requires to initiate an immune reaction (36). The meticulous design of epitopes is a pivotal step in targeted therapies and crucial for minimizing potential harm to healthy cells. AI algorithms play a significant role in this design process. AI-powered approaches have significantly enhanced the accuracy of forecasting epitopes for designing TCVs by considering the relative affinities of adjacent amino acids (37). Over the past few decades, researchers have developed numerous AI algorithms for epitope design and the prediction of MHC peptide binding, as depicted in **Table 2**. Since the indicators are diverse, they are elaborated upon in the supplementary paper. It enumerates a variety of studies at the intersection of AI and cancer vaccine development. These studies employ various AI techniques, such as neural networks (e.g., BepiPred and MHCflurry-2), support vector machines (SVMs) (e.g., DeepImuno and Epitopia), and natural language processing (e.g., MHCSeqNet), to predict and assess critical aspects of cancer vaccines, including epitope binding, immunogenicity, and antigen presentation. While each tool has its unique strengths and limitations, collectively, they emphasize the transformative potential of AI in enhancing our understanding of cancer immunology and the finding of effective neoantigens for TCVs development.

**Table 2:** A Comprehensive Assessment of Studies for the Development of Cancer Vaccines Using AI Techniques.

Reference	Purpose	Approach	Dataset Used	Performance evaluation metrics	Contribution	Limitation
(47)	To forecast the likelihood of peptides reaching the cell surface and activating T cells.	Recurrent Neural Network (RNN)	32,785 peptides for the forecast of immunogenicity and 437,077 peptide sequences for the binding mechanism.	Accuracy = 0.9	Both the potential for HLA-peptide interaction and the model for the immunogenicity of the peptide-MHC (pMHC) complex were considered when predicting neoantigens.	The confined set of immune-stimulating pMHC combinations can challenge the accuracy of immunogenicity models.
(48)	To determine T cell epitopes, naturally occurring ligands, and cancer neoantigens.	NN, Artificial Neural Networks (ANNs).	Studies of affinity from IEDB & and 85,217 peptides	Specificity (98.5%)	The methods performance extended to a wider range of MHC molecules. It surpassed the relatively small number of MHC molecules data during the training.	It made use of training data that favors peptides with lengths shorter than nine.
(49)	To forecast Presented Peptides of MHC Class I.	NN’s	A total of 493,473 MS records and 219,596 affinity measures made up the training set.	AUC= 0.90	An integrated prediction of MHC class I presentation was created by combining fresh models for antigen processing and MHC binding.	Training data for the predictor model contain both antigen processing-sensitive and insensitive data. So, the ranking of strong binders may not accurately reflect antigen processing signals.
(50)	To find neoantigen candidates that have fewer false positives, which can be utilized to assess the patient’s prognosis.	Gaussian naıve Bayes (GNB), locally weighted naıve Bayes (LNB), random forest (RF), and SVM.	There are 311 neoepitopes eliciting a T-cell response.	Accuracy = 0.981	It can be utilized to evaluate the patient’s prognosis and identify neoantigen candidates with fewer false positives.	This model’s drawback is that a single value assigned to a discrete aspect of the entire process is insufficient to resolve the level of complexity.
(51)	To predict the T cell receptor (TCR) binding specificities of class I pMHC complexes presenting neoantigens and T cell antigens in general.	Long short-term memory (LSTM)	243,747 human TCR CDR3 sequences, 172,422 affinity measurements, and 32,607 pMHC-TCR pairs.	AUC = 0.827	Based on the TCR sequence, neoantigen sequence, and MHC type, this model allowed for the forecasting of the TCR-binding specificity of class I pMHCs.	The drawback of pMTnet is that it can only forecast using certain types of epitopes, MHCs, and TCRs.
(52)	To locate neo-epitopes in cancer immunotherapy and pathogen epitopes in infectious diseases.	Logistic Regression	4958 peptides	Accuracy = 0.75	They increased the predictability of immunogenic CD8+ T-cell epitopes by coupling antigen presentation with peptide-intrinsic TCR detection propensity.	Its performance is not as good when integrated with other predictors as it is when utilized independently.
(53)	To precisely forecast whether HLA-II complexes will exhibit a given peptide.	RNN, LSTM	33,909 peptides were used for the binding model, 12,150 for the cleavage model, and 8374 for the presentation model.	Accuracy = 0.89-0.92	This tool makes it possible to find immunogenic epitopes in various malignancies and autoimmune conditions.	When employed as cancer vaccines, numerous peptides with high presentation scores failed to elicit CD4+ responses.
(54)	To identify useful neoepitopes for the development of TCVs	NLP, GRU, NN	228,348 peptides	Accuracy = 0.92	It assisted in the selection of useful neoepitopes for the creation of cancer vaccines.	Because there isn’t enough training data or for other reasons, they might not support all MHC alleles.
(55)	To improve the accuracy of predictions made on multiple separate test sets for both conformational and linear epitope prediction.	FFNN (Feed Forward), CNN, and LSTM, Random Forest Classifier (RFC)	BP3 and BP3C50ID	Accuracy = 0.762	An epitope prediction tool based on sequence that can predict both linear and conformational epitopes.	The BepiPred-3.0 model currently in use relies on known solved antibody-antigen complexes, which only make up a tiny portion of all potential pathogenic proteins and antibodies.
(56)	To predict linear antigenic epitopes.	SVM	The dataset consisted of 65,456 B-cell linear epitopes from IEDB.	Sensitivity – 80.1% Precision – 55.2% AUC - 0.70	A web service that integrates tripeptide similarity and propensity scores from a human protein sequence backdrop to predict non-redundant B-cell linear epitopes.	The performance of SVMTriP in predicting discontinuous epitopes is not stated.
(57)	To develop a web server for B-cell conformational epitope prediction.	RNN	Discotope dataset and the Epitome dataset	Accuracy = 0.75	A software suite used for protein structure prediction improves upon the constraints of earlier predictors by integrating an amino-acid propensity scale, alongside side chain orientation and solvent accessibility information, utilizing half-sphere exposure values.	The focus on linear B-cell epitopes, as opposed to discontinuous epitopes, which make up a significant fraction of B-cell epitopes, is a shortcoming of this ML approach.
(58)	To identify areas of a protein sequence and structure that are immunogenic.	Naïve Bayes, SVM	194 non-redundant epitopes from antigen sequences and 66 non-redundant epitopes from antibody-antigen co-crystal structures were used to train the model.	AUC – 0.53 for the CEP method	A web-based application capable of predicting immunogenic regions in a protein, whether in its 3D structure or linear sequence.	It relies on a benchmark dataset of well-known epitopes.
(59)	To use sequence alone to determine the immunogenic potential of peptides	ElasticNet, K-nearest neighbors (KNN), SVM, RF, and AdaBoost.	IEDB dataset	AUROC = 0.85	An online interface for forecasting the potential immunogenicity of specific HLA alleles, as well as identifying which patients could gain the most from immunogenic therapy.	This model’s weakness is that it has a lower specificity than is ideal for confidently choosing immunogenic antigens.

One of the challenges in identifying neoantigens is forecasting the peptides that can bind to MHC class I molecules and be presented on the surface of melanoma cells. Various computational techniques have been employed to address this problem, using ML algorithms and mass spectrometry (MS) data. ML-based methods rely on large training datasets of known MHC class I-bound peptides, which are often limited and incomplete. Therefore, these methods may miss some neoantigens that are not well-represented in the databases. To overcome this limitation, Abelin et al. (38) used MS to profile the MHC ligandomes of cells expressing single HLA-I alleles and identified 24,000 peptides presented by these alleles. They also examined the impact of protein cleavage and gene expression levels on antigen presentation. MS-based methods can also be used to create more accurate and comprehensive training datasets for ML-based methods. For example, a recent method used MS to collect 185,000 high-quality MHC class I-bound peptides from human tumor biopsies and used them to train an ML model that achieved over 75% prediction accuracy (39). Another approach is to use a consortium of different ML models to improve prediction performance (40). Racle et al. (41) created MoDec, a motif deconvolution algorithm similar in concept to convolutional neural networks (CNNs), using mass spectrometry-based peptide datasets. This algorithm aims to identify MHC II-binding motifs by incorporating core offset preferences and peptide cleavage motifs. They had a dataset comprising 23 different samples and 77,189 unique peptides in total. These methods showcase the synergy between ML and MS in enhancing the identification of neoantigens, potentially resulting in more effective and personalized cancer immunotherapy.

Vaccine development relies heavily on computational prediction of T-cell epitopes, focusing on antigen processing and presentation mechanisms such as transporter associated with antigen processing (TAP), proteasomal cleavage, and MHC binding. Developing computational techniques that are accurate, rapid, and capable of providing comprehensive insights into this binding can significantly accelerate the progress of immunotherapies and the development of vaccines in general. A crucial step in targeting melanoma cells is binding off these MHC molecules to the epitope and presentation to cytotoxic T-cells. In this context, Chu et al. (42) introduced the TransMut framework, consisting of TransPHLA for pHLA binding prediction and Adaptive Optimization of Mutated Peptides (AOMP) for modified peptide optimization. TransPHLA, powered by a self-attention model derived from Transformers, excels in predicting pHLA binding, neoantigens, and the Human Papillomavirus Vaccine (HPV) identification, outperforming 13 other methods.

One of the most used techniques for MHC peptide binding prediction and epitope design is SVMs (43). They are employed to predict T-cell epitopes by utilizing data descriptions to determine peptide binding preferences. For example, Zhao et al. (44) developed an SVM model for peptide-MHC molecule interaction. The foundation of developing novel immuno-diagnostic reagents and vaccines is the identification of protein surface areas that antibodies preferentially recognize (antigenic epitopes). Computational techniques for antigenic epitope identification offer essential tools to support this endeavor. Tri-peptide similarity and propensity scores have been combined to use SVMTriP. Using a five-fold cross-validation, SVMTriP obtains a sensitivity of 80.1% and a precision of 55.2% when applied to non-redundant B-cell linear epitopes retrieved from IEDB. The AUC (Area Under the Curve) results in a value of 0.702. The accuracy for linear B-cell epitopes can be enhanced by combining the similarity and propensity of tripeptide sequences An SVM-based technique was developed by Nagpal et al. (45) to forecast peptides’ capacity to alter APCs for antigen presentation. Their developed modulator demonstrated an impressive accuracy rate of 95.71% on the training dataset and has been made publicly accessible via a web-based server named VaxinPAD. The effectiveness of these technologies highlights the importance of continued innovation and precision in computational techniques to facilitate advancements in this field. Accurately predicting peptides that bind to MHC class I molecules and are subsequently expressed on melanoma cells using AI methods exhibits several significant challenges. Firstly, the limited amount and variability of experimental data, especially for rare MHC alleles, hinders the training and effectiveness of AI models. Secondly, the vast diversity of MHC alleles in the human population, each with unique binding preferences, necessitates pan-specific models capable of generalizing across this complexity (46). Further compounding the issue is the complex and dynamic nature of peptide processing and presentation, influenced by various cellular factors. Finally, selecting the most suitable AI model from a plethora of available options requires careful consideration of data type, features, parameters, and performance metrics to ensure optimal prediction accuracy. Addressing these challenges is crucial for harnessing the full potential of AI in developing effective immunotherapy strategies against melanoma.

### 5.2 Advancements in AI-driven cancer diagnosis and personalized treatment strategies

Biomarkers are measurable signals or indicators used to assess various biological processes, including the presence of cancerous cells or the effectiveness of a specific treatment (60). One critical aspect of AI-driven cancer vaccines involves mutation and biomarker prediction, enabling the identification of specific genetic alterations and biomarkers for accurate cancer diagnosis and personalized therapies. Cancer biomarkers are biomolecules created by the body or within a tumor in individuals with cancer. These biomarkers encompass various forms, including DNA, RNA, proteins, or metabolic patterns, and are unique to the tumor (61). By scrutinizing unique genetic or molecular alterations inherent to a patient’s specific tumor, medical practitioners can precisely determine the most appropriate treatment strategies. Significantly, in research centered on breast cancer, the identification of the HER2 biomarker has played a crucial role in recognizing individuals who can gain advantages from targeted therapies like trastuzumab (62). Through HER2 biomarker testing, clinicians can gauge the likelihood of a patient’s cancer responding positively to this specific treatment, thereby avoiding ineffective therapies and possible adverse effects. Following the identification of these biomarkers, the next phase focuses on determining whether there are exploitable genetic alterations that accelerate tumor development.

These biomarkers are classified into various groups according to their nature and function. Genetic Biomarkers involve the analysis of specific genetic mutations or alterations associated with cancer (63). Protein biomarkers involve the measurement of specific proteins or protein expressions associated with cancer (64). These biomarkers can be used to predict treatment response or monitor disease progression. For example, the prostate-specific antigen (PSA) is commonly used to monitor treatment response and detect recurrence in prostate cancer (65). Imaging biomarkers utilize various imaging techniques, such as MRI or PET scans, to assess tumor characteristics or treatment response (66). These biomarkers provide valuable information about tumor size, location, and metabolic activity, aiding in treatment planning and evaluation. Liquid biomarkers involve the analysis of various substances present in body fluids (67). These biomarkers can provide non-invasive and real-time information about tumor characteristics or treatment response. For example, circulating tumor nucleic acids (DNA) in the blood can help in monitor treatment response and detect minimal residual disease. In this context, Wood et al. (68) developed a fully automated, AI-driven somatic mutation finding tool called Cerebro. The model employs an RF algorithm to assess numerous decision trees, generating confidence scores for potential mutations. Cerebro demonstrated superior performance in identifying validated mutations, with high sensitivity (97%) and positive predictive value (98%).

Mutations in the Isocitrate dehydrogenase (IDH) gene is an important biomarker for the diagnosis and management of gliomas (a type of brain tumor) (69). Using tumor slides with Hematoxylin & Eosin (H&E) stains from glioma patients, Liu et al. (70) suggested a GAN-based data enhancement technique to improve the prediction of these mutations. Through data augmentation, their study greatly improved prediction accuracy. In the context of cutaneous melanoma, a promising mRNA-based biomarker signature has been developed by Bai et al. (71). The signature, created through a combination of Cox proportional hazards regression and random survival forest algorithms, outperformed clinical prognostic markers and holds significant potential to be a predictive biomarker for cutaneous melanoma. Classifying cancers based only on mutations is challenging due to intratumor heterogeneity, low tumor purity, and common mutations across cancer types. A greater comprehension of the spatial connection between immune and other cells in individual tissues is made possible by the clinical use of contemporary analytical and diagnostic tools like multiplexed immune- and genetic analysis (72, 73) along with AI. This reveals relevant intratumor heterogeneity, which may have significant ramifications for immune-related and combination therapies (74, 75). Despite the burgeoning arsenal of immunotherapies, their successful integration into clinical practice hinges upon a thorough understanding established through academic research, economic viability, and demonstrably positive clinical outcomes (76–78).

Genomic profiles obtained from alternative sources like cell-free DNA (cfDNA) can provide valuable insights into the genetic landscape of tumors and aid in the classification of cancers. ML can be crucial in analyzing complex genomic data (79) and improving our ability to classify and understand cancer types. AI predictive models have shown promising potential in identifying specific mutations or genetic characteristics related to imaging symptoms in various medical conditions, including cancer. In this context, Mu et al. (80) developed a DL model based on PET/CT images to distinguish between patients with EGFR mutations and wild-type non-small cell lung carcinoma (NSCLC) patients, achieving an accuracy of 0.81. This model leveraged radiometric characteristics for accurate prediction. DeepVariant (81) uses DCNNs to find small indels in sequencing data. In the PrecisionFDA Truth Challenge, it outperformed other variant callers with remarkable accuracy. The technology can assist in identifying neoantigens or indels that cause cancer. Advanced methods like Fusion-Bloom (82) offer improved fusion variant detection through the application of a structural mutation detection technique based on transcriptome assembly. The algorithm has demonstrated higher specificity and sensitivity in detecting true fusion variants. These AI-based tools for mutation and biomarker prediction are making significant strides in advancing cancer diagnosis and personalized treatment strategies. These developments hold great promise for improving the quality of life for cancer patients.

### 5.3 AI in immunogenicity prediction

Vaccine immunogenicity refers to the extent to which a vaccine can trigger the immune system to initiate an immune response. Immunogenicity prediction helps in streamlined vaccine development, reduced costs, enhanced safety assessment, optimized dosing strategies, tailored vaccine designs for specific populations, and rapid response. An innovative method for determining a peptide’s immunogenic potential based just on its sequence was presented by Li et al. (59). They introduced a beta-binomial distribution method and evaluated its performance against other models. Surprisingly, the CNN emerged as the most important prediction model due to its adaptability for datasets of different sizes. Additionally, the team introduced an independent GAN called DeepImmuno-GAN. This innovative approach successfully replicated immunogenic peptides, aligning their physicochemical properties and immunogenicity predictions to real antigens.

Diao et al. (83) created a model using CNN called Seq2Neo-CNN to forecast the immunogenicity of peptides. The model’s performance was evaluated in comparison with other ML models (SVM, random forest, ExtraTree, logistic regression, and XGBoost), which were also trained using data from the independent TESLA dataset. This model achieved an accuracy rate of 0.801. Numerous other examples exist, such as, Wang et al. (84) created INeo-Epp, a classifier based on random forest for predicting the immunogenicity of T-cell epitopes, including neoantigens (14). discussed the overall composition, organization, and activity of immune cells within a specific tissue on clinical response and the potential use of ML in finding new T-cell neoepitope. The 3T-TRACE platform developed by 3T biosciences utilizes active ML with extensive target libraries to discover new targets and T-cell receptors (TCRs). This method identifies the most common and immunogenic targets within solid tumors and is suitable for all tumor types (85). Not all MHC-presented peptides provoke an immune response. myNEO ImmunoEngine, a personalized cancer platform developed by myNEO, also emphasizes structural attributes when predicting the immunogenicity of neoantigens (86).

While AI-driven models have shown significant promise in predicting immunogenicity, several challenges remain, particularly concerning tumor heterogeneity and individual genetic variability. Tumor heterogeneity—wherein distinct subclones with varying mutational burdens and immune-evasive strategies exist within the same tumor—presents a substantial hurdle (87, 88). AI models may accurately predict neoantigens for one subclone but fail to account for others, leading to incomplete or ineffective immune responses. This limitation highlights the necessity for more dynamic, real-time data integration into AI models to capture evolving tumor profiles. Moreover, individual genetic variability, especially within the Major Histocompatibility Complex (MHC), significantly influences immune responses (88). AI models trained on data from common MHC alleles may not generalize well to individuals with rare alleles, resulting in suboptimal vaccine designs for certain populations. This underscores the importance of training AI models on diverse datasets that encompass a broader range of genetic backgrounds, allowing for more personalized and effective vaccine development.

Vaccine allergenicity refers to the potential of a vaccine to trigger an allergic reaction in the recipients. For allergenicity prediction, Dimitrov et al. (89) introduced AlgPred and AllerTOP 1.0 servers for assessing the allergenicity of chimeric proteins. These servers offer multiple ways to determine allergenicity, including using allergen representative peptides (ARPs) and hybrid methods. It entails searching a database of 2890 ARPs acquired from Gasteiger et al. (90) for the query protein sequence. The hybrid option combines an alignment-free method based on the primary physicochemical properties of proteins with KNN classification, achieving a high sensitivity of 94% (89). For antigenicity assessment, two servers, ANTIGENpro and VaxiJen v2.0 (91), were employed. ANTIGENpro, an alignment-free server, based on sequence, uses microarray data on protein antigenic properties to predict protein antigenicity (92). Conversely, VaxiJen v2.0 achieves an 89% classification accuracy by classifying antigens only based on the physicochemical characteristics of proteins (93). These diverse applications of AI in immunogenicity prediction and related fields showcase the vast potential of AI in advancing our understanding and treatment of cancer. These studies collectively illustrate the use of AI techniques to enhance the precision and effectiveness of immunogenicity prediction, paving the way for more targeted and effective therapeutic interventions.

## 6 Utilizing AI in the vaccine design

The integrated use of AI in cancer vaccine development is multifaceted, encompassing several critical processes. Firstly, AI plays a crucial role in target identification and validation. It analyzes extensive genomic and molecular data to pinpoint potential tumor antigens, thereby significantly optimizing the selection of immune system targets. This allows for a more precise and focused approach to immunotherapy. Secondly, AI contributes to vaccine design and optimization. By simulating interactions with the immune system, AI can predict vaccine efficacy and guide researchers in choosing the most promising candidates for further development. This significantly improves the efficiency and effectiveness of the vaccine creation process. Categorically, cancer vaccines materialize as four distinct types: tumor or immune cell-based, viral vector-based, peptide-based, and nucleic acid-based (94). Therapeutic Cancer Vaccines (TCVs) aim to activate the adaptive immune system, coordinating a coordinated attack against specific tumor antigens (95). By orchestrating these mechanisms, TCVs aim to train the immune system to identify and eliminate cancerous cells, providing a targeted and specific approach to cancer immunotherapy. TCVs often aim to induce a Th1 immune response, characterized by the activation of killer T cells and the release of pro-inflammatory cytokines. This response is particularly effective in combating cancer cells. **Table 3** offers an overview of multiple companies involved in clinical trials for cancer vaccines, with a common theme being the integration of AI technology in their development pipelines. These trials encompass a range of cancer types and innovative projects.

**Table 3:** Clinical studies for an AI-powered cancer vaccine.

Reference	Company	Tool Name	Type of Vaccine	Vaccine Name	Type of cancer	Remarks	Trial Number
(165)	Moderna/Merck & co.	–	mRNA vaccine	mRNA-4157	High-risk melanoma	Phase-II trial	NCT03897881
(166)	Moderna and IBM	–	mRNA vaccine	KRAS vaccine (mRNA-5671)	Lung, Pancreatic, and Colon cancer	Phase-I trial	NCT03948763
(167)			mRNA vaccine	Checkpoint vaccine (mRNA-4359)	Advanced solid tumor	Phase-I trial	NCT05533697
(101)	Evaxion	PIONEER & OBsERV	Peptide vaccine	EVX-01	Metastatic or Unresectable Melanoma	Phase-II trial	NCT05309421
(168)	Nykode	NeoSELECT	DNA vaccine	VB10.16	HPV-positive cancers	–	NCT06099418
(169)	BioNTech	Protein design tool DeepChain	mRNA-based cancer vaccine	BNT-122	Localized or Metastatic Prostate Cancer	Phase-II trial	NCT04486378
(170)	Transgene and NEC	Their technology is driven by NEC’s AI capabilities and the myvac platform.	DNA-based vaccine	TG4050	Head and neck cancer	Phase-I trial	NCT04183166
(171)	Columbia University	GeneWays	Peptide vaccine	NY-ESO-1 combined with MPLA	Lung, ovarian and melanoma cancer	Phase I/II	NCT01584115
(172)	University of Virginia	Deep Neural Net QSAR	Peptide vaccine	LPV7	Mucosal and metastatic Melanoma	Phase I/II	NCT02126579
(173)	Wakayama Medical University	DeepTox	peptide-based vaccine	URLC10-177 and TTK-567, two tumor-specific epitope peptides, combined with CpG7909	Esophageal	Phase I/II	NCT00669292

### 6.1 AI in nucleic acid vaccine design

#### 6.1.1 DNA vaccines

These are plasmids expressed by bacteria containing DNA sequences that encode antigenic proteins. These vaccines demonstrate the potential to elicit strong immune responses, thereby aiding in the fight against cancer. An ideal example is the ongoing clinical trial (phase-I) for breast cancer involving a Mammaglobin-A DNA vaccine (NCT00807781) (96). Another case in point is the clinical effectiveness demonstrated by a DNA vaccine targeting HPV-16/HPV-18 E6 and E7 oncogenes in individuals with high-grade cervical intraepithelial neoplasia (97). DNA vaccines offer several advantages, including ease of manufacturing, inherent adjuvants, and a good source of TAA. Nevertheless, they require additional steps of transcription and translation before dendritic cells can cross-present them for immune activation (18). There are apprehensions regarding the possible integration of the DNA vaccine into the host genome, which may result in unintended outcomes, including the activation of oncogenes or interference with regular cellular functions (98). The immune system might perceive the DNA vector as foreign, eliciting an immune reaction against it. This response has the potential to diminish the vaccine’s effectiveness and trigger adverse reactions (99).

Efforts are being made to increase the immunogenicity of DNA vaccines to improve their effectiveness. Choosing and fine-tuning the optimal antigens for incorporation into the plasmid DNA is a strategy to enhance immune responses induced by the vaccine and improve therapeutic effectiveness (100). Evaxion Biotech’s cancer vaccine, EVX-01, represents an enhanced, advanced iteration of DNA-based neoantigen cancer immunotherapy for metastatic melanoma, developed to address advanced solid cancers (101). Their AI platform, PIONEER, helps create neoantigens for targeted cancer therapy (102). Immunoinformatics can aid in identifying tumor-specific antigens that are likely to trigger rapid and protective immune responses for DNA vaccines. AI algorithms can enhance these immunoinformatic approaches (103). Lurescia et al. (104) discuss the use of immunoinformatics in designing DNA vaccines against B-cell lymphoma. Combining AI with genome editing via CRISPR/Cas9 presents a novel frontier in precise gene mutation modification, molecular cloning, and tumor genome alteration (105). This synergy expedites gene editing processes by harnessing AI’s analytical capabilities to interpret data and construct knowledge models (106). Genetic vaccines are made possible by CRISPR-Cas, which delivers certain antigen-encoding genes into host cells to stimulate a strong and focused immune response. These developments in CRISPR-based immune augmentation are very promising for treating cancer, infectious illnesses, and other situations where effective treatment depends on a strong immune system (107).

As this field advances, there is a growing focus on enhancing poly epitope DNA constructs for clinical application, requiring thorough validation of modifications and epitope combinations through experimental predictions and *in vitro* preclinical investigations (108). To maximize efficacy, it is essential to concentrate on strategies that enhance epitope expression, fine-tune the recruitment of the immune system, and identify the most suitable combinations of epitopes. By utilizing predictive algorithms, exome/genome sequences, RNA sequencing analysis, and comparative sequencing of a patient’s tumor and standard samples, it becomes feasible to pinpoint tumor neoantigens. A vaccine can be designed to trigger an immune reaction targeted at the specific neoantigens identified, with minimal risk of provoking tumor growth (109). Absci, a biotech company, has developed a model called CO-BERT of codon optimization using advanced DL algorithms to enhance the process, specifically focusing on predicting the most effective codons for achieving maximum protein expression (110). This innovative approach allows them to design gene sequences with precise modulation for heightened expression within a particular host organism, circumventing the traditional trial-and-error method in the laboratory. This advancement bears significance in scenarios such as DNA vaccine development, facilitating optimal antigen expression and eliciting an intensified immune response within the body. With ongoing advancements, the horizon of DNA-based cancer vaccines appears promising, enabling tailored treatments based on individual MHC/TAA epitope profiles.

#### 6.1.2 RNA vaccines

These are popularly known as mRNA vaccines, which contain a small piece of synthetic mRNA that codes for either TAAs or neoantigens (111). Like DNA vaccines, RNA vaccines offer the advantage of being relatively simple to produce and having inherent adjuvant properties (112). Furthermore, with the growing emphasis on tumor mutational burden and the potential benefits of personalized vaccines targeting neo-epitopes (113), significant progress has been made in developing vaccination strategies for cancer treatment. However, unlike DNA vaccines, RNA vaccines bypass the need for transcription, allowing for faster presentation by MHC molecules. This streamlined process enhances their efficiency in triggering immune responses. A recent study has demonstrated the potent ability of RNA vaccines to elicit robust immune responses against cancer (114), further supporting their potential as an effective strategy in cancer immunotherapy. AI technology plays a crucial role in optimizing mRNA vaccine structures, enhancing their immunogenicity, safety, and overall efficacy (111). It can also assist in designing nanoparticles for self-assembling mRNA vaccines, improving their stability and delivery (115).

Researchers can use DL techniques to optimize mRNA sequences to enhance protein expression and antigen presentation, producing a more focused and potent immune response (116). Through computer modeling supported by ML, scientists can simulate the interactions and reactions of the components of the immune system in response to diseases like cancer, both in the presence and absence of vaccines (117). This approach assists in the development of effective mRNA sequences for vaccination purposes. Understanding the secondary structure of RNA molecules is crucial for gaining insights into their cellular functions, and designing of novel drugs and vaccines. In this context, Singh and colleagues (118) introduce an innovative DL-based approach for predicting RNA secondary structures. Other tools, such as SPOT-RNA, DMfold, and CDPfold, specialize in predicting mRNA stability, structure, and binding (119). RNA degradation is a crucial process that affects the expression and function of genes. Predicting the degradation features of mRNA sequences, such as the half-life and the degradation rate at each nucleotide, can provide insights into the regulation and dynamics of gene expression. However, this task is challenging due to the complex interactions between RNA molecules and various factors that influence their stability. He et al. (120) proposed a CNN model called RNA deformer, which utilizes self-attention to recognize both local and global dependencies in RNA sequences. The convolution layer can extract local features such as secondary structures, while the self-attention layer can learn long-range dependencies and interactions. The model also provides interpretability by visualizing the attention weights of the self-attention layer, which can reveal the importance of different regions and nucleotides for degradation prediction. The authors demonstrated that their model achieved high accuracy and outperformed existing methods on two datasets: the OpenVaccine dataset, which contains mRNA sequences of COVID-19 vaccine candidates, and the m6A-modified dataset, which contains mRNA sequences with m6A modifications. Their results showed that fine-tuning mRNA degradation and half-life is necessary for the safety, efficacy, and proper functioning of mRNA vaccines. Human Immunology Project Consortium (HIPC) (https://www.immuneprofiling.org), researchers can perform transcriptional profiling analyses of the immune system (including microRNA arrays, and next-generation sequencing (121)) before and after a particular infection, vaccination, or adjuvant treatment (122). This will enable a more comprehensive assessment of the efficacy and safety of different vaccine formulations, as well as expedite the evaluation of human disease.

By creating a valuable tool for tissue collection, *in vitro* tumor culture, and medicine screening, the integration of AI into organoids is expected to tackle the safety and customization issues associated with traditional prediction (123). By concentrating on antigen presentation pathways (124) and cell necroptosis index (CNI) (125), AI could also predict ICB responses. Using a multiplex gene detection method, Mizukami et al.’s systems biology approach to vaccine safety assessment and the discovery of certain indicators in a rat preclinical investigation allowed for the evaluation of vaccine safety against the pandemic H5N1 influenza (126). It’s worth noting that numerous in silico models are currently employed to predict protein-protein and drug-protein interactions based on available datasets. This suggests that AI models hold potential for simulating interactions between adjuvants and vaccines. This capability extends beyond cancer vaccines, as there are existing adjuvant databases that can be leveraged for other vaccine development purposes as well (127). For instance, using DL, researchers have modeled the association between human 5′ UTR sequences and mRNA translation, offering insights into potential translation efficiencies (128). CNNs excel in identifying motifs in DNA or mRNA sequences and have demonstrated superior performance compared to previous non-DL methods in the domain, as seen in convolution-based architectures such as deep learning-based sequence models (129). However, CNNs encounter challenges in capturing distant relationships, which are vital for tasks involving DNA and RNA. AI algorithms, like transformers, have shown promise in predicting sequences of DNA and RNA (130, 131). These approaches can help optimize vaccine development and enhance the understanding of molecular processes. Leading companies like Moderna are at the forefront of mRNA vaccine technology and rely heavily on AI for numerous aspects of their research processes. They are also developing three types of mRNA vaccines for cancer: mRNA-4157, mRNA-5671, and mRNA-4359 (132). Furthermore, continued research is required to improve the accuracy of AI-guided predictions and to fine-tune the techniques used in preparing these vaccines.

### 6.2 Peptide vaccines

Peptide-based cancer vaccines leverage the immune system to initiate a targeted response against tumor-associated antigens, ultimately leading to the destruction of cancer cells. However, one of the major challenges faced in designing effective cancer vaccines is the intra-tumoral heterogeneity, which refers to the significant variability in genetic and phenotypic traits among cancer cells within a single tumor (133). This variability results in a diverse population of cells, some of which may evade immune detection or develop resistance to treatments.

The immune system plays a critical role in cancer immunosurveillance by identifying and eliminating transformed cells to prevent tumor formation (134). Yet, tumors characterized by pronounced heterogeneity often display complex interactions among various subclones, leading to fluctuations in immune cell infiltration and activation. This dynamic environment can result in subclones that respond differently to immune-based therapies, thereby complicating the effectiveness of immunotherapy approaches (135).

Addressing this challenge requires a multi-faceted strategy that can account for the diverse subpopulations of cancer cells. Stephens et al. (136) highlighted the significance of crafting sophisticated peptide-based cancer vaccines designed to specifically target and activate various components of the immune system, particularly antigen-presenting cells. A promising approach is the development of personalized peptide vaccines based on neoantigens (137). These vaccines aim to boost the immune system’s ability to fight tumors by using neoantigens, which are unique to each patient’s cancer (138).

Developing peptide-based vaccines is complex due to the diversity of MHC alleles in the human population. AI models used to predict T-cell epitopes can have limitations, as the spatial configuration of epitopes changes when antigens bind to cell surface receptors, leading to potential false-positive and false-negative results (139). Additionally, MHC-II-restricted peptides are highly diverse and challenging to predict due to their complexity compared to MHC-I cells (140). Advancements in bioinformatics tools, including predicting HLA allele coverage and using promiscuous peptides that bind to multiple MHC alleles, offer potential solutions to address the challenges of peptide vaccine design (141). One significant obstacle is the enormous variation in MHC-I molecules among people. It is difficult to predict which peptides will attach to a particular patient’s MHC-I because there are thousands of distinct MHC-I alleles (142). Immunodominance presents another difficulty since not all tumor antigens are equally effective at eliciting a robust immune response. The peptides’ structural integrity is also very important, as it influences their ability to interact with T cell receptors and initiate an effective immune response (143). It’s possible that the peptides won’t be sufficient to trigger a strong immunological response. To get around this, scientists are looking into the use of adjuvants, which are substances that strengthen the immune system and increase the efficacy of vaccinations. Another obstacle that must be tackled involves the creation of adjuvants and efficient delivery methods to ensure the peptides reach their intended destinations effectively (144).

Automated ML systems like SIMON (Sequential Iterative Modeling “OverNight”) compare results from diverse clinical datasets, improving predictive accuracy and providing new vaccine targets (145). AI models such as RF, SVM, Recursive Feature Selection, Deep Convolutional Neural Networks (DCNN), LSTM networks, NEC Immune Profiler, and the Immune Epitope Database (IEDB) assist in predicting epitopes and designing effective peptide vaccines (137). ML is also being employed in the design of peptide-based nanomaterials for tumor immunotherapy (146). Also, AI can offer solutions to improve the affinity and stability of Peptide-based inhibitors for immune checkpoint blockade (ICB) and make them more effective in clinical settings (147). In conclusion, peptide-based cancer vaccines have the potential to be a powerful tool in the fight against cancer. However, there are still challenges in antigen selection, adjuvant use, and personalized approaches. Companies like Ardigen are developing AI tools like ARDesign and the ARDitox platform to help design peptide-based cancer vaccines (148). AI is emerging as a valuable tool to address the challenges of peptide vaccine design and improve its efficacy in cancer immunotherapy.

The ARDesign platform developed by Ardigen can identify targets for TCR-based therapeutics and design both customized and off-the-shelf Personalized Cancer Vaccines (PCVs). It consists of the following models: ARDitox, ARDisplay (presentation model), Ardimmune (immunogenicity model), and Meta-model (which takes into account metrics and their output). As one of the inputs for the ARDesign platform, DNA sequences will be compared to the standard genome, and both somatic and germline mutations will be identified (149). Cross-reactivity between certain TCRs and substantially different epitope sequences has been reported. Sanecka-Duin et al. (150) developed ARDitox, an advanced in silico technique for detecting and evaluating potential off-target binding, utilizing computational immunology and artificial intelligence (AI) to address this issue. They utilized a set of TCRs targeting a viral epitope and numerous cases from the literature where TCRs were employed to target cancer-relevant antigens to assess the efficacy of ARDitox in silico.

### 6.3 Dendritic cell vaccines

DCVs are activated dendritic cells that are exposed to cancer antigens. They are infused into patients to elicit an immune response against melanoma cells (151). The components used in DCVs consist of antigens obtained from the patient’s tumor cells, and in some cases, subsets such as cancer stem cells can also be utilized (152). Acquisition of these cells can be achieved through methods like biopsy or surgical extraction of the tumor. The selected antigens are typically proteins that demonstrate distinctive or elevated expression levels in cancer cells compared to normal cells. In the laboratory, dendritic cells are then subjected to these extracted antigens. This exposure process may entail co-culturing dendritic cells with tumor cell lysates or purified antigens (153, 154). emphasized the difficulties in extrapolating immune responses from small trials to large trials and the preference for autologous tumor lysate-based strategies rather than shared antigens. DCVs target tumor-specific peptides or epitopes to induce anti-tumor effects. These epitopes are short amino acid sequences derived from TAAs and chosen for their immunogenicity and compatibility with HLA alleles. ANNs have been used to estimate the binding strength between MHC molecules and different positions of peptide sequences (115). Mirsanei et al. (155)used an ANN mathematical model to improve DCV delivery. Their research aimed to identify the optimal DC dosage and administration schedule to enhance the effectiveness of DC-based immunotherapy. ANNs can process data on nano-bio interactions and predict optimal DC vaccination parameters, such as DC types, activation techniques, and injection sites.

Creating superior personalized DCVs involves considering various factors, including individual patient physiological information, nanoparticle features, and tumor variation (156). Paulis et al. (157) explored the design of DC-based nano vaccines for tumor immunotherapy, emphasizing the delivery of cancer antigens and immunostimulatory signals to elicit potent anti-tumor responses. Hashemi et al. (158) focused on using nanoparticles for targeted drug delivery to DCs, aiming to improve the effectiveness and stability of immunological reactions in DC-based cancer immunotherapy. Nanoparticles (NPs) are essential for promoting strong T-cell and B-cell immune reactions, increasing the absorption by cells of immunostimulatory representatives, and frequently acting as self-adjuvants. Despite these advantageous attributes, a comprehensive exploration of the interactions between these naturally derived NPs and diverse biological components, including immune cells, remains imperative. This thorough investigation is essential for evaluating immunotoxicity and propelling the progress of immunostimulatory NPs as a secure and efficacious tool in the realm of cancer immunotherapy (159). Scientists have created AI algorithms capable of forecasting the ideal dimensions, structure, and surface properties of nanoparticles designed for drug delivery and cancer immunotherapy. For example, an Artificial Neural Network (ANN) was utilized to predict the size and initial release rates of poly (lactic-co-glycolic acid) (PLGA) nanoparticles (160).

NPs vary according to the tumor model, cancer type, and physicochemical characteristics. Development and research on cancer nanomedicine can benefit from the integration of ML/AI with physiologically based pharmacokinetic (PBPK) models, as demonstrated by Lin et al. (161). Their DL model can serve as a platform to aid in the design of future cancer nanomedicines and help scientists decide which NPs should proceed to pre-clinical trials, thereby reducing and enhancing animal investigations. When designing nanostructures and RNA nano designs, computer-aided and mathematical modeling can assist in selecting specific building blocks to achieve desired structures (162). By utilizing ML and AI simulations, the design of nanoparticles can facilitate the understanding of molecular interactions. This computer-aided approach enhances comprehension of RNA and nanoparticle interactions, thereby increasing the likelihood of success and optimizing nanoparticle utilization.

Because of the interactions with small molecules, peptides, receptors, antigens, and nucleic acids, nanoparticles in particular are helpful in medical biophysics. Particularly, gold nanoparticles (Au NPs) hold great promise for drug delivery, cancer therapy, and therapeutic and diagnostic applications. Owing to their optimal dimensions, form, and surface area, Au NPs are adaptable elements that offer encouraging outcomes and support. Optimal therapeutic conditions are ensured by their perfect cellular absorption and preventative cytotoxicity measures. Through control using AI and mathematical modeling, Au NPs have advanced and will continue to progress in medical biophysics. This topical review emphasizes the significance of advancing future nanotechnology research through the integration of mathematical modeling and artificial intelligence (AI) to enhance medical biophysics (163). Suberi et al. (164) developed an image-based technique to enhance vaccine manufacturing by incorporating image-based techniques that require minimal computational time for analysis and investigation. Incorporating AI into the development and optimization of dendritic cell-based cancer vaccines holds promise for enhancing their effectiveness and personalization. AI-driven analyses can help identify optimal peptide sequences, vaccination parameters, and vaccine manufacturing processes, contributing to more effective cancer immunotherapy (18).

## 7 Adjuvant development and AI

Adjuvants are molecules with the ability to enhance and/or shape antigen-specific immune responses (144). Adjuvants, such as oil-in-water mixtures and aluminum salts, have been in use for years (174). However, many adjuvants face challenges during development, such as stability, efficacy, tolerability, or safety concerns. For instance, aluminum salts can induce antigen aggregation, thereby influencing the stability and immunogenicity of vaccines (175). Squalene-based adjuvants, like MF59 and AS03, have been associated with injection site reactions (176). Recent advancements in AI have resulted in the discovery of two novel, broad-spectrum adjuvants through computer-aided molecular design and ML.

While adjuvants have been integral components of vaccinations for an extended period, their precise mechanisms of action and their effects on enhancing or altering immune responses triggered by vaccinations are often unclear. The model vaccine utilized in this study was a Self-Assembling Protein Nanoparticle (SAPN) displaying the malarial circumsporozoite protein (CSP), adjuvanted with three distinct liposomal formulations: liposome plus Alum (ALFA), liposome plus QS21 (ALFQ), and both (ALFQA). They identified unique vaccine-induced immune responses by using a computational approach to combine the immune-profiling data. They also constructed a multivariate model that was able to forecast the adjuvant condition with 92% accuracy based solely on immune response data. This served as an effective means of locating putative immunological correlates of protection, which is necessary to match vaccination candidates with adjuvants in a logical manner (177). In contrast, AI is capable of quickly and effectively analyzing enormous databases of molecular and genetic data to locate possible adjuvants that might boost the immune system. Moreover, AI systems can potentially simulate the interactions between adjuvants and immune cells, allowing researchers to optimize adjuvant doses and combinations. This optimization leads to the generation of an ideal immunological response, making the growth of more effective and tailored cancer treatments possible.

Adjuvants play a crucial role in shaping vaccine-induced immune responses through a variety of intricate yet often subtle mechanisms, underscoring their indispensable contribution to the efficacy of vaccinations. Adjuvant-specific immune response features may be identified by machine learning and in-depth analysis of vaccine-induced cytokine, cellular, and antibody responses (known as “immune profiling”). This information could be utilized to make informed decisions regarding the rational selection of adjuvants. Chaudhury et al. (178) investigated the profiles of human immune responses elicited by vaccines adjuvanted with two comparable, clinically significant adjuvants, AS01B and AS02A. They identified important differentiators, or immunological signatures, that these adjuvants imprint on vaccine-induced immunity. The computational analysis identified a combination of immunological characteristics that could classify participants by adjuvant with 71% accuracy. Additionally, it revealed statistically significant changes in cellular and antibody responses between cohorts.

Adjuvants stimulate a strong immunological response in response to a vaccination (77). Despite being in use for decades, many adjuvants used today, like oil-in-water emulsions and aluminum salts, do not produce widespread or durable immune responses. Stronger adjuvants are therefore required. Through the utilization of machine learning and computer-aided molecular design, Ma et al. (179) discovered two novel broad-spectrum adjuvants with the potential to augment vaccine responses. Their library includes 46 toll-like receptor (TLR)–targeting agonist ligands that were synthesized on Au nanoparticles. This study illustrates computer-aided design and testing that can rapidly identify effective adjuvants to counteract the declining immunity associated with current vaccinations. AI offers a promising avenue for accelerating adjuvant development in cancer vaccines. It streamlines the identification of effective adjuvants, optimizes their usage, and enhances our ability to create more efficient and individualized cancer therapies while saving valuable time and resources.

## 8 Personalized cancer vaccines

Personalized cancer vaccines represent a promising strategy to enhance the efficacy of immunotherapy by specifically targeting cancer-associated antigens. In a pivotal study, Ott et al. (180) demonstrated that tailored neoantigen therapy, combined with the checkpoint inhibitor anti-PD-1, was both well-tolerated and effective in patients with advanced tumors, such as non-small cell lung cancer and bladder cancer. Similarly, in the Phase I GAPVAC-101 study conducted by the Glioma Actively Personalized Vaccine Consortium, Hilf et al. (181) integrated highly personalized vaccines containing both tumor-specific and shared antigens into standard therapies. This approach aimed to maximize the therapeutic use of the limited target space available for individuals diagnosed with newly diagnosed glioblastoma.

Neoantigens, which arise from protein-coding mutations specific to tumors, play a crucial role in eliciting robust immune responses (182, 183). These neoantigens can serve as potent targets for cancer vaccines, aiding in the rejection of tumors (94). For tumors with typically "cold" immunological microenvironments, such as glioblastoma, a personalized neoantigen vaccination strategy using multiple epitopes has shown feasibility. This approach has also been explored in high-risk melanoma patients (184). Keskin et al. (185) further demonstrated that neoantigen-specific T cells derived from peripheral blood could infiltrate an intracranial glioblastoma tumor, as revealed through single-cell T-cell receptor research. Sahin et al. (186) reported that personalized RNA mutanome vaccines, either as a standalone therapy or in combination with anti-PD-L1, triggered multi-specific therapeutic immune responses, resulting in objective clinical improvements in some patients with advanced tumors. These findings underscore the potential of personalized cancer vaccines to improve outcomes for patients across various cancer types. 

AI-driven progress is set to be a key factor in the emerging area of personalized tumor vaccines, signifying the forefront of innovation. A crucial initial step in developing customized TCVs is the identification of tumor-specific neoantigens (TSNs) present on the exterior of cancer cells. The incorporation of AI into this process significantly accelerates the identification and selection of potential cancer vaccines for individual patients. By utilizing machine learning (ML) to translate somatic mutations into actionable neoantigens, AI offers more effective selection of immune responses with the greatest therapeutic potential (187, 188). Additionally, other computational approaches, such as combining genetic algorithms with support vector machines (SVMs), have achieved high predictive accuracy in vaccine development (43). One such tool, PREDIVAC (189), excels at identifying CD4+ T-cell epitopes, surpassing existing methods in predicting HLA class II peptide binding. Integrating computational analysis, high-throughput genomics, and machine learning can significantly streamline the identification of therapeutic targets and response biomarkers, which are critical for the development of effective personalized cancer vaccines. However, achieving success in this domain will require the development of interpretable AI systems capable of explaining the rationale behind their conclusions, ensuring transparency and trust in AI-assisted decision-making processes.

## 9 Open challenges for developing cancer vaccines using AI

The critical importance of large, high-quality datasets in training AI models is underscored by the need for accurate and relevant information. To develop robust models capable of handling noisy data and generalizing to untrained samples, it is essential to ensure that the data feeding these models is both comprehensive and precise. This becomes particularly important when predicting B-cell epitopes, which vary widely in location, size, and sequence within proteins. The challenge of predicting antigenic epitopes lies in identifying regions of a protein that can bind to antibodies, a task that becomes even more complex when considering entire protein complexes. Predicting antigenic epitopes in a multi-chain protein structure requires modeling interactions between the chains, adding layers of complexity compared to single-chain protein predictions. Moreover, antigenic epitopes are subject to selective pressure from host immunity, making them more variable than standard binding sites. This variability renders antigenic epitope prediction poorly suited for traditional binding site prediction techniques (190). Comparatively, several conformational B-cell epitope-forecasting algorithms, which employ ML techniques alongside features like conservation, structure, geometry, and amino acid properties, have outperformed binding site prediction models in terms of accuracy. 

However, even with these advancements, the presence of clonal diversity and intra-tumor heterogeneity in both primary and metastatic tumors presents a significant challenge to mutation-dependent neoantigen predictions (191, 192). Not all neoantigens can serve as viable targets for cancer vaccines, highlighting the difficulty in identifying immunologically relevant neo-peptides. For cancers with a lower mutational burden, the need for mutation-independent neoantigens becomes even more critical. Unfortunately, most current AI models primarily focus on mutation-dependent neoantigen prediction, often overlooking important factors such as tumor accessibility (193). A crucial aspect of neoantigen prediction is the role of HLA alleles, which present antigens to the immune system. Accurate neoantigen prediction must consider the alignment of peptide mutations with the patient's unique HLA alleles (194). By incorporating this relationship, researchers can more effectively identify neoantigens capable of eliciting a robust immune response, which is essential for developing personalized cancer immunotherapies (195, 196). The integration of these features into prediction algorithms has the potential to significantly enhance their performance (197).

T-cell migration within the tumor microenvironment may encounter obstacles, including the extracellular matrix and Cancer-related macrophages, which can constrain T-cell access to tumor antigens (198). Tumor accessibility, affected by factors such as poor vascularization, physical barriers, and the presence of blood vessels limiting T-cell infiltration, constitutes a critical stage following the generation of neoantigen-specific T-cell responses (199), yet this aspect is often overlooked in current prediction tools due to dataset limitations. Single-cell analysis can be used to address bulk sequencing’s shortcomings in capturing the heterogeneity of the tumor immune microenvironment (200).

Vaccine efficiency is also influenced by T-cell inability to detect immune-evading tumors and T-cell suppression by the immunosuppressive tumor microenvironment (TME) (201). Efforts to model TME have been made (202), but current AI models struggle to detect immune-evading tumors. Personalized neoantigen cancer vaccines also face challenges with T-cell exhaustion and dysfunction, branded by a loss of effecter function, enhanced repressive receptor expression, and a tendency for cell death, significantly limiting their benefits (199). Early predictive signatures for vaccine responses are critical for advancing next-generation cancer vaccines, necessitating ongoing research.

## 10 Future prospects

Future research in cancer vaccine design should prioritize strategies aimed at enhancing the recruitment of immune cells, increasing epitope expression, and selecting optimal combinations of epitopes capable of eliciting robust T-cell responses against tumor-associated antigens. Advances in molecular sequencing, AI, and cellular engineering hold the potential to revolutionize cancer vaccines, making them faster, more affordable, and more effective. These technologies allow for rapid and comprehensive assessment of immune responses to cancer vaccinations, facilitating real-time adjustments based on individual patient responses. Digitally transformed clinical studies play a crucial role in predicting patient reactions under different conditions, leading to more precise vaccine development. The application of ML and stochastic optimization techniques contributes to identifying optimal vaccination strategies, ensuring efficacy with minimal doses. AI technologies can forecast potential changes in melanoma cells, enabling the development of vaccines that remain effective against driver mutations. Moreover, AI can collaborate with systems like VARES (Vaccine Adverse Event Reporting System) to identify populations that could face potential vaccination risks. In addition to predictive modeling, AI models and synthetic developmental biology have led to the creation of biobots and xenobots with potential applications in cancer therapy. These innovative entities could play roles ranging from drug delivery to tumor targeting, expanding the scope of cancer treatment possibilities. The synergy between AI, molecular advancements, and biobots represents a multifaceted approach to advancing cancer vaccine design and delivery. AI facilitates real-time adjustments based on individual patient responses, predictive modeling, and the identification of optimal vaccination strategies. However, ethical and legal considerations, such as data privacy, algorithm bias, regulatory compliance (203), and equitable access, are of utmost importance.

Nano computers are devices that control or guide nanobots inside the body. They can be electrical, biological, organic, or quantum in nature. Software programmed using four nitrogenous base letters from DNA can control the expression of genes in computers built at the molecular level from DNA. It can also identify the type of mRNA associated with specific genes that, when overexpressed or conversely under-expressed, can contribute to cancer development. This makes it possible to diagnose various cancer forms and treat the illness with the recommended medication (204). In 2016, researchers developed simulated nanobots designed to target and destroy brain cancer cells (205). These nanobots possess the capability to recognize and eradicate cancerous cells. Upon detection of the tumor, they emit an auditory signal, facilitating precise localization for subsequent intervention. The advent of artificial intelligence (AI) has raised numerous legal and ethical dilemmas for society, encompassing concerns related to privacy and surveillance, bias and discrimination, and the role of human judgment. These challenges may also present philosophical complexities. The emergence of newer digital technologies has sparked concerns that they could introduce additional sources of errors and data breaches. In the medical field, errors in processes or protocols can have catastrophic consequences for patients who fall victim to such mistakes.

Finding ethical and legal issues could also be a proactive way to reduce risks, which would help AI technology become more widely used and successful overall. Transparency in the application of AI technology can be fostered by resolving ethical concerns and guaranteeing regulatory compliance, which can improve patient trust—a crucial component of healthcare. Another potential aspect in assessing the effectiveness of immunotherapy is AI’s ability to identify lymphocytes, tumor cells, and mesenchymal stroma in the slice and to emphasize the spatial distribution of various cell types using 3-D modeling (206). For immunotherapy, an optimal AI-based predictive model should incorporate all pertinent clinical data about the patient, such as genetics, imaging, proteomics, pathological tissue, demographic data, medical history, etc. To allow the sharing of huge data from many locations, it is vital to enhance the integrity and objectivity of data gathering since ideas such as pan-cancer analysis have been reflected in the assessment of PD-1/PD-L1 efficacy (207). This bodes very well for immunotherapy’s future (208).

Clinicians are beginning to pay more and more attention to DL neural networks, as they demonstrate good performance in tracking and predicting treatment responses (209). Nowadays, solid tumor diagnosis (tumors of the gastrointestinal tract, lung cancer, melanoma, etc.) is accomplished using biomarker analysis, computerized histopathological image interpretation, or automatic quantification of radiological imaging (210). Nevertheless, there hasn’t been much study on using AI to assess the effectiveness of immunotherapy in NSCLC (Non-small cell lung cancer). Russo et al. (203)’s computational framework has the potential to expedite and improve the process of designing and developing the optimal vaccine formulation that can boost the immune system’s response, hence boosting the efficiency of therapeutic vaccines and protecting prophylactic ones. With the use of in silico trial technology, the advantages of this computational pipeline may expedite and improve the design of phase I/II clinical trials for the most promising vaccine candidates that have been found. There is a lot of work being done for IST to receive the regulatory bodies’ qualifying stamp.

Currently, healthcare settings lack clear legislation to address the ethical and legal concerns that may arise from the utilization of artificial intelligence. Striking a balance between innovation and ethical safeguards is crucial to ensuring the responsible development and deployment of AI-driven cancer vaccines.

### 10.1 Summary

Cancer vaccine research is focused on improving immune responses through enhanced recruitment and epitope selection. Advanced technologies like molecular sequencing, artificial intelligence (AI), and cellular engineering hold promise for faster and more effective vaccine development. AI enables real-time adjustments based on individual patient responses, while Nano computers and nanobots offer innovative drug delivery solutions. However, ethical and legal considerations, including data privacy and algorithm bias, must be addressed to ensure responsible deployment of AI-driven cancer vaccines. Balancing innovation with ethical safeguards is crucial for safe and effective cancer treatment.

## 11 Discussion

Despite attempts to compile a diverse range of studies, the review acknowledges the limitations arising from data availability primarily in the English language. This linguistic limitation raises awareness of the potential exclusion of valuable insights from non-English literature. This underscores the importance of future reviews to adopt a more encompassing language approach. The use of PRISMA-ScR methodology is employed to mitigate personal biases in data collection. Furthermore, the disclaimer stating that the review paper is not accountable for the results of the cited research papers raises questions about the reliability and validity of the included studies. This prompts a consideration of the methodological rigor of the underlying research and its implications on the overall robustness of the review’s findings. Furthermore, the deliberate emphasis on high-quality papers from reputable journals introduces a potential bias that should be acknowledged when interpreting the conclusions of the review. This underscores the importance of having a nuanced understanding of the limitations associated with excluding research from lesser-known journals.

The review significantly contributes by highlighting the complex challenges in developing effective cancer vaccines, covering areas such as target identification, immunological tolerance, and patient-specific variations. It guides researchers through this intricate landscape. The review also plays a crucial role in explaining fundamental biological concepts and the application of AI tools in cancer vaccine design. The study explores biomarker prediction, epitope design, MHC binding prediction, and immunogenicity prediction, bridging traditional biology with advanced AI applications. The detailed exploration of various cancer vaccine types, including DNA, mRNA, peptide, and dendritic cell vaccines, enhances comprehension. The inclusion of tables summarizing datasets, AI techniques in vaccine development studies, and ongoing clinical trials serves as a valuable resource for researchers, facilitating data gathering and comparative analysis. These tables also offer insights into the current research landscape and guide future research efforts. Beyond its specific contributions, the review initiates a broader conversation about the challenges impeding the broad adoption of cancer vaccines. It delves into reasons for their limited usage, examining challenges in clinical adoption, and regulatory aspects, providing context to the current status of therapeutic cancer vaccine development. The review also extends its focus beyond vaccine design to explore AI’s potential in designing clinical trials, finding people with potential vaccination risks, and selecting optimum dosage. This wider viewpoint emphasizes the transformative impact of AI, not just in vaccine development but also in reshaping different aspects of cancer treatment, including patient stratification and personalized therapeutic strategies.

## 12 Conclusion

The advent of cancer vaccines represents a significant stride in global cancer treatment. Leveraging AI and ML-based techniques during vaccine development enables the precise prediction and identification of neoantigens capable of triggering robust anti-tumor immune responses. Recent progress in cancer vaccine discovery has partially addressed the challenges associated with antigen selection. These novel approaches incorporate diverse mechanisms to counteract cancer cells’ immune-suppressive effects, paving the way for personalized cancer vaccine development. AI has been pivotal in tailoring these vaccines, presenting a promising solution to the limitations of conventional therapies. For optimal outcomes, deploying cancer vaccines early in the disease’s progression or during its minimal residual stages appears to be most effective. Advances in related fields like artificial intelligence, cellular technology, and DNA sequencing could benefit these vaccines, all of which facilitate the examination and optimization of vaccine-induced immunological reactions. While computational technologies for designing nucleic acid vaccines, such as epitope prediction and sequence optimization, currently demonstrate moderate performance levels due to evolving technology, the availability of extensive online data and advanced models holds significant promise. ML algorithms can predict epitopes and have integrated into several prediction approaches, albeit with some accuracy limitations. One of the difficulties is the restricted supply of high-quality data for developing and evaluating AI models in a clinical setting. Although the creation of synthetic immunogenic peptide models via GANs and diffusion models remains relatively unexplored, these tools have been successful in domains like computer vision and synthetic biology, generating novel images and sequences of interest. The potential of AI to tailor treatments for individual cancer patients and expedite the development of new technologies promises to revolutionize the field of oncology therapy. Immunomics is poised to take a leading role in the future of tumor immunology but there is still a substantial amount of work to be done. Advances in AI, especially in immunogenomics and single-cell analysis, are expected to significantly advance this field’s clinical utility. Greater accessibility to data with experimental validation and the ongoing development of improved algorithms, driven by extensive datasets, will enhance vaccine design strategies.

While AI presents a promising avenue for accelerating cancer vaccine design, it is essential to acknowledge its limitations, particularly regarding the complexity of biological systems and the challenges of clinical translation. AI-driven models, despite their predictive power, are often constrained by the availability and quality of data, and their predictions may not fully account for the dynamic nature of the immune system and tumor microenvironment. Biological factors such as immune evasion, tumor heterogeneity, the unpredictable nature of immune responses and individual genetic variability can significantly impact the effectiveness of vaccines in clinical settings. Thus, AI should be viewed as a complementary tool that works alongside traditional experimental approaches rather than as a standalone solution. Experimental validation, animal models, and rigorous clinical trials remain crucial.

In conclusion, the AI-driven cancer vaccine design and development is gaining significant attention in academia and industry. Various companies are contributing substantial resources to the field to address current challenges, offering customized solutions using AI. The vital role of therapeutic cancer vaccines is highlighted by developments in tumor biology and vaccine technology, especially in cases of early-stage or minimal residual disease. The progress in the development of tumor vaccines is driven by the critical demand for effective cancer treatments. These advancements could greatly benefit from collaborative efforts across multiple disciplines, including immunology and cancer biology, as well as the integration of artificial intelligence (AI).

## Appendix

**Table A1:** List of all the abbreviations used in this manuscript, accompanied by the meaning of each one.

Acronym	Definition
AI	Artificial Intelligence
ANN	Artificial Neural Network
AOMP	Adaptive Optimization of Mutated Peptide
APCs	*Antigen-Presenting Cell*
AUC	*Area Under the Curve*
CNN	Convolutional Neural Networks
DC	Dendritic Cells
DCV	Dendritic Cell Vaccines
DL	Deep Learning
DNA	Deoxyribonucleic acid
DNN	Deep Neural Network
EGFR	Estimated Glomerular Filtration Rate
GAN	Generative Adversarial Network
GNN	Graph Neural Network
HLA	Human Leukocyte Antigen
HPV	Human Papillomavirus Vaccine
IDH	Isocitrate Dehydrogenase
LSTM	Long Short-Term Memory
MHC	Major Histocompatibility Complex
ML	Machine Learning
MOA	Mechanism-Of-Action
MRI	*magnetic resonance imaging*
mRNA	*messenger ribonucleic acid*
MS	Mass Spectrometry
NN	Neural Network
NSCLC	Non-Small Cell Lung Carcinoma
PAMPs	Pathogen-Associated Molecular Patterns
PET scan	positron Emission Tomography
PLGA	Poly (Lactic-co-Glycolic Acid)
PRRs	Pattern Recognition Receptors
PSA	Prostate-Specific Antigen
RF	Random Forest
RNA	*Ribonucleic acid*
SVM	Support Vector Machines
TAP	Tumor Antigen Processing
TAA	Tumor-Associated Antigens
TCRs	T-Cell Receptors
TCV	Therapeutic Cancer Vaccines
TLR	Toll-Like Receptors
TME	Tumor Micro-Environment
TSNs	Tumor-Specific Neoantigens
VARES	Vaccine Adverse Event Reporting System

## References

[1] Cai Y Chen R Gao S Li W Liu Y Su G. Artificial intelligence applied in neoantigen identification facilitates personalized cancer immunotherapy. Front Oncol. (2023) 12:1054231. doi: 10.3389/fonc.2022.1054231 36698417 PMC9868469

[2] Sung H Ferlay J Siegel RL Laversanne M Soerjomataram I Jemal A. Global cancer statistics 2020: GLOBOCAN estimates of incidence and mortality worldwide for 36 cancers in 185 countries. CA Cancer J Clin. (2021) 71:209–49. doi: 10.3322/caac.21660 33538338

[3] Vishweshwaraiah YL Dokholyan NV. mRNA vaccines for cancer immunotherapy. Front Immunol. (2022) 13:1029069. doi: 10.3389/fimmu.2022.1029069 36591226 PMC9794995

[4] Miao L Zhang Y Huang L. mRNA vaccine for cancer immunotherapy. Mol Cancer. (2021) 20:41. doi: 10.1186/s12943-021-01335-5 33632261 PMC7905014

[5] Faghfuri E Pourfarzi F Faghfouri AH Abdoli Shadbad M Hajiasgharzadeh K Baradaran B. Recent developments of RNA-based vaccines in cancer immunotherapy. Expert Opin Biol Ther. (2021) 21:201–18. doi: 10.1080/14712598.2020.1815704 32842798

[6] Jia Q Wang A Yuan Y Zhu B Long H. Heterogeneity of the tumor immune microenvironment and its clinical relevance. Exp Hematol Oncol. (2022) 11:24. doi: 10.1186/s40164-022-00277-y 35461288 PMC9034473

[7] Monie A Hung C-F Roden R Wu T-C. Cervarix: a vaccine for the prevention of HPV 16, 18-associated cervical cancer. Biologics. (2008) 2:97–105. doi: 10.2147/BTT.S1877 PMC2727782 19707432

[8] McLemore MR. Gardasil®: introducing the new human papillomavirus vaccine. Clin J Oncol Nurs. (2006) 10:559–60. doi: 10.1188/06.CJON.559-560 17063609

[9] Kirby T. FDA approves new upgraded Gardasil 9. Lancet Oncol. (2015) 16:e56. doi: 10.1016/S1470-2045(14)71191-X 25532625

[10] Zhao H Zhou X Zhou Y-H. Hepatitis B vaccine development and implementation. Hum Vaccin Immunother. (2020) 16:1533–44. doi: 10.1080/21645515.2020.1732166 PMC7482909 32186974

[11] Ullah M Akbar A Yannarelli G. Applications of artificial intelligence in, early detection of cancer, clinical diagnosis and personalized medicine. WArtificial Intell Cancer. (2020) 1:39–44. doi: 10.35713/aic.v1.i2.39

[12] Kringelum JV Lundegaard C Lund O Nielsen M. Reliable B cell epitope predictions: impacts of method development and improved benchmarking. PloS Comput Biol. (2012) 8:e1002829. doi: 10.1371/journal.pcbi.1002829 23300419 PMC3531324

[13] Vaka AR Soni B K. SR. Breast cancer detection by leveraging Machine Learning. ICT Express. (2020) 6:320–4. doi: 10.1016/j.icte.2020.04.009

[14] Xu Z Wang X Zeng S Ren X Yan Y Gong Z. Applying artificial intelligence for cancer immunotherapy. Acta Pharm Sin B. (2021) 11:3393–405. doi: 10.1016/j.apsb.2021.02.007 PMC8642413 34900525

[15] Kohn MS Sun J Knoop S Shabo A Carmeli B Sow D. IBM’s health analytics and clinical decision support. Yearb Med Inform. (2014) 23:154–62. doi: 10.15265/IY-2014-0002 PMC4287097 25123736

[16] Høie MH Gade FS Johansen JM Würtzen C Winther O Nielsen M. DiscoTope-3.0: improved B-cell epitope prediction using inverse folding latent representations. Front Immunol. (2024) 15:1322712. doi: 10.3389/fimmu.2024.1322712 38390326 PMC10882062

[17] Waltz E. AI takes its best shot: What AI can—and can’t—do in the race for a coronavirus vaccine - [Vaccine. IEEE Spectr. (2020) 57:24–67. doi: 10.1109/MSPEC.2020.9205545

[18] Saxena M van der Burg SH Melief CJM Bhardwaj N. Therapeutic cancer vaccines. Nat Rev Cancer. (2021) 21:360–78. doi: 10.1038/s41568-021-00346-0 33907315

[19] Oth T Vanderlocht J Van Elssen CHMJ Bos GMJ Germeraad WTV. Pathogen-associated molecular patterns induced crosstalk between dendritic cells, T helper cells, and natural killer helper cells can improve dendritic cell vaccination. Mediators Inflammation. (2016) 2016:1–12. doi: 10.1155/2016/5740373 PMC4766350 26980946

[20] Alberts B Johnson A Lewis J Raff M Roberts K Walter P. Molecular Biology of the Cell, 4th edition. (New York: Annals of Botany) (2017) 91.

[21] Wang S Liu H Zhang X Qian F. Intranasal and oral vaccination with protein-based antigens: advantages, challenges and formulation strategies. Protein Cell. (2015) 6:480–503. doi: 10.1007/s13238-015-0164-2 25944045 PMC4491048

[22] Macri C Dumont C Johnston AP Mintern JD. Targeting dendritic cells: a promising strategy to improve vaccine effectiveness. Clin Transl Immunol. (2016) 5:e66. doi: 10.1038/cti.2016.6 PMC4815026 27217957

[23] Birkholz K Schwenkert M Kellner C Gross S Fey G Schuler-Thurner B. Targeting of DEC-205 on human dendritic cells results in efficient MHC class II–restricted antigen presentation. Blood. (2010) 116(23):2277–85. doi: 10.1182/blood-2010-02-268425 20566893

[24] Caminschi I Proietto AI Ahmet F Kitsoulis S Shin Teh J Lo JC. The dendritic cell subtype-restricted C-type lectin Clec9A is a target for vaccine enhancement. Blood. (2008) 112(8):3264–73. doi: 10.1182/blood-2008-05-155176 PMC2569177 18669894

[25] Macri C Dumont C Panozza S Lahoud MH Caminschi I Villadangos JA. Antibody-mediated targeting of antigen to C-type lectin-like receptors Clec9A and Clec12A elicits different vaccination outcomes. Mol Immunol. (2017) 81:143–50. doi: 10.1016/j.molimm.2016.12.010 27978488

[26] Fucikova J Kepp O Kasikova L Petroni G Yamazaki T Liu P. Detection of immunogenic cell death and its relevance for cancer therapy. Cell Death Dis. (2020) 11(11):1013. doi: 10.1038/s41419-020-03221-2 33243969 PMC7691519

[27] Garg AD Agostinis P. Cell death and immunity in cancer: From danger signals to mimicry of pathogen defense responses. Immunol Rev. (2017) 280:126–48. doi: 10.1111/imr.12574 29027218

[28] Schuler MM Nastke M-D Stevanović S. SYFPEITHI. Methods Mol Biol. (2007) 409:75–93. doi: 10.1007/978-1-60327-118-9_5 18449993

[29] Schisler NJ. The IDB and IEDB: intron sequence and evolution databases. Nucleic Acids Res. (2000) 28:181–4. doi: 10.1093/nar/28.1.181 PMC102469 10592220

[30] Lu M Xu L Jian X Tan X Zhao J Liu Z. dbPepNeo2.0: A database for human tumor neoantigen peptides from mass spectrometry and TCR recognition. Front Immunol. (2022) 13:855976. doi: 10.3389/fimmu.2022.855976 35493528 PMC9043652

[31] Lata S Bhasin M Raghava GP. MHCBN 4.0: A database of MHC/TAP binding peptides and T-cell epitopes. BMC Res Notes. (2009) 2:61. doi: 10.1186/1756-0500-2-61 19379493 PMC2679046

[32] Kardani K Bolhassani A. Cppsite 2.0: an available database of experimentally validated cell-penetrating peptides predicting their secondary and tertiary structures. J Mol Biol. (2021) 433:166703. doi: 10.1016/j.jmb.2020.11.002 33186582

[33] Almeida LG Sakabe NJ deOliveira AR Silva MC Mundstein AS Cohen T. CTdatabase: a knowledge-base of high-throughput and curated data on cancer-testis antigens. Nucleic Acids Res. (2009) 37:D816–9. doi: 10.1093/nar/gkn673 PMC2686577 18838390

[34] Fang LT Afshar PT Chhibber A Mohiyuddin M Fan Y Mu JC. An ensemble approach to accurately detect somatic mutations using SomaticSeq. Genome Biol. (2015) 16:197. doi: 10.1186/s13059-015-0758-2 26381235 PMC4574535

[35] Bulik-Sullivan B Busby J Palmer CD Davis MJ Murphy T Clark A. Deep learning using tumor HLA peptide mass spectrometry datasets improves neoantigen identification. Nat Biotechnol. (2019) 37(1):55–63. doi: 10.1038/nbt.4313 30556813

[36] Kogay R Schönbach C. Epitope predictions. In: Encyclopedia of Bioinformatics and Computational Biology. Elsevier (2019). p. 952–71. doi: 10.1016/B978-0-12-809633-8.20248-3

[37] Cherryholmes GA Stanton SE Disis ML. Current methods of epitope identification for cancer vaccine design. Vaccine. (2015) 33:7408–14. doi: 10.1016/j.vaccine.2015.06.116 26238725

[38] Abelin JG Keskin DB Sarkizova S Hartigan CR Zhang W Sidney J. Mass spectrometry profiling of HLA-associated peptidomes in mono-allelic cells enables more accurate epitope prediction. Immunity. (2017) 46(2):315–26. doi: 10.1016/j.immuni.2017.02.007 PMC5405381 28228285

[39] Sarkizova S Klaeger S Le PM Li LW Oliveira G Keshishian H. A large peptidome dataset improves HLA class I epitope prediction across most of the human population. Nat Biotechnol. (2020) 38(2):199–209. doi: 10.1038/s41587-019-0322-9 31844290 PMC7008090

[40] Wells DK van Buuren MM Dang KK Hubbard-Lucey VM Sheehan KC Campbell KM. Key parameters of tumor epitope immunogenicity revealed through a consortium approach improve neoantigen prediction. Cell. (2020) 183(3):818–834.e13. doi: 10.1016/j.cell.2020.09.015 33038342 PMC7652061

[41] Racle J Michaux J Rockinger GA Arnaud M Bobisse S Chong C. Robust prediction of HLA class II epitopes by deep motif deconvolution of immunopeptidomes. Nat Biotechnol. (2019) 37(11):1283–6. doi: 10.1038/s41587-019-0289-6 31611696

[42] Chu Y Zhang Y Wang Q Zhang L Wang X Wang Y. A transformer-based model to predict peptide–HLA class I binding and optimize mutated peptides for vaccine design. Nat Mach Intell. (2022) 4(3):300–11. doi: 10.1038/s42256-022-00459-7

[43] Moghram BA Nabil E Badr A. Ab-initio conformational epitope structure prediction using genetic algorithm and SVM for vaccine design. Comput Methods Programs BioMed. (2018) 153:161–70. doi: 10.1016/j.cmpb.2017.10.011 29157448

[44] Zhao Y Pinilla C Valmori D Martin R Simon R. Application of support vector machines for T-cell epitopes prediction. Bioinformatics. (2003) 19:1978–84. doi: 10.1093/bioinformatics/btg255 14555632

[45] Nagpal G Chaudhary K Agrawal P Raghava GPS. Computer-aided prediction of antigen presenting cell modulators for designing peptide-based vaccine adjuvants. J Transl Med. (2018) 16:181. doi: 10.1186/s12967-018-1560-1 29970096 PMC6029051

[46] Rasmussen M Fenoy E Harndahl M Kristensen AB Nielsen IK Nielsen M. Pan-specific prediction of peptide-MHC class I complex stability, a correlate of T cell immunogenicity. J Immunol. (2016) 197(4):1517–24. doi: 10.4049/jimmunol.1600582 PMC4976001 27402703

[47] Wu J Wang W Zhang J Zhou B Zhao W Su Z. DeepHLApan: A deep learning approach for neoantigen prediction considering both HLA-peptide binding and immunogenicity. Front Immunol. (2019) 10:2559. doi: 10.3389/fimmu.2019.02559 31736974 PMC6838785

[48] Jurtz V Paul S Andreatta M Marcatili P Peters B Nielsen M. NetMHCpan-4.0: improved peptide–MHC class I interaction predictions integrating eluted ligand and peptide binding affinity data. J Immunol. (2017) 199:3360–8. doi: 10.4049/jimmunol.1700893 PMC5679736 28978689

[49] O’Donnell TJ Rubinsteyn A Laserson U. MHCflurry 2.0: improved pan-allele prediction of MHC class I-presented peptides by incorporating antigen processing. Cell Syst. (2020) 11:42–48.e7. doi: 10.1016/j.cels.2020.06.010 32711842

[50] Kim S Kim HS Kim E Lee MG Shin EC Paik S. Neopepsee: accurate genome-level prediction of neoantigens by harnessing sequence and amino acid immunogenicity information. Ann Oncol. (2018) 29(4):1030–6. doi: 10.1093/annonc/mdy022 29360924

[51] Lu T Zhang Z Zhu J Wang Y Jiang P Xiao X. Deep learning-based prediction of the T cell receptor–antigen binding specificity. Nat Mach Intell. (2021) 3(10):864–75. doi: 10.1038/s42256-021-00383-2 PMC9396750 36003885

[52] Schmidt J Smith AR Magnin M Racle J Devlin JR Bobisse S. Prediction of neo-epitope immunogenicity reveals TCR recognition determinants and provides insight into immunoediting. Cell Rep Med. (2021) 2(2):100194. doi: 10.1016/j.xcrm.2021.100194 33665637 PMC7897774

[53] Chen B Khodadoust MS Olsson N Wagar LE Fast E Liu CL. Predicting HLA class II antigen presentation through integrated deep learning. Nat Biotechnol. (2019) 37(11):1332–43. doi: 10.1038/s41587-019-0280-2 PMC7075463 31611695

[54] Phloyphisut P Pornputtapong N Sriswasdi S Chuangsuwanich E. MHCSeqNet: a deep neural network model for universal MHC binding prediction. BMC Bioinf. (2019) 20:270. doi: 10.1186/s12859-019-2892-4 PMC6540523 31138107

[55] Clifford JN Høie MH Deleuran S Peters B Nielsen M Marcatili P. BepiPred -3.0: Improved B-cell epitope prediction using protein language models. Protein Sci. (2022) 31:e4497. doi: 10.1002/pro.4497 36366745 PMC9679979

[56] Yao B Zhang L Liang S Zhang C. SVMTriP: A method to predict antigenic epitopes using support vector machine to integrate tri-peptide similarity and propensity. PloS One. (2012) 7:e45152. doi: 10.1371/journal.pone.0045152 22984622 PMC3440317

[57] Sweredoski MJ Baldi P. PEPITO: improved discontinuous B-cell epitope prediction using multiple distance thresholds and half sphere exposure. Bioinformatics. (2008) 24:1459–60. doi: 10.1093/bioinformatics/btn199 18443018

[58] Rubinstein ND Mayrose I Martz E Pupko T. Epitopia: a web-server for predicting B-cell epitopes. BMC Bioinf. (2009) 10:287. doi: 10.1186/1471-2105-10-287 PMC2751785 19751513

[59] Li G Iyer B Prasath VBS Ni Y Salomonis N. DeepImmuno: deep learning-empowered prediction and generation of immunogenic peptides for T-cell immunity. Brief Bioinform. (2021) 22:bbab160. doi: 10.1093/bib/bbab160 34009266 PMC8135853

[60] Califf RM. Biomarker definitions and their applications. Exp Biol Med. (2018) 243:. 213–221. doi: 10.1177/1535370217750088 PMC5813875 29405771

[61] Henry NL Hayes DF. Cancer biomarkers. Mol Oncol. (2012) 6:140–6. doi: 10.1016/j.molonc.2012.01.010 PMC5528374 22356776

[62] Mercogliano MF Bruni S Mauro FL Schillaci R. Emerging targeted therapies for HER2-positive breast cancer. Cancers (Basel). (2023) 15:1987. doi: 10.3390/cancers15071987 37046648 PMC10093019

[63] Herceg Z Hainaut P. Genetic and epigenetic alterations as biomarkers for cancer detection, diagnosis and prognosis. Mol Oncol. (2007) 1:26–41. doi: 10.1016/j.molonc.2007.01.004 19383285 PMC5543860

[64] Yang D-W Zhang Y Hong QY Hu J Li C Pan BS. Role of a serum-based biomarker panel in the early diagnosis of lung cancer for a cohort of high-risk patients. Cancer. (2015) 121:3113–21. doi: 10.1002/cncr.29551 26331818

[65] Lilja H Ulmert D Vickers AJ. Prostate-specific antigen and prostate cancer: prediction, detection and monitoring. Nat Rev Cancer. (2008) 8:268–78. doi: 10.1038/nrc2351 18337732

[66] Waldman AD Jackson A Price SJ Clark CA Booth TC Auer DP. Quantitative imaging biomarkers in neuro-oncology. Nat Rev Clin Oncol. (2009) 6(8):445–54. doi: 10.1038/nrclinonc.2009.92 19546864

[67] Heitzer E Haque IS Roberts CES Speicher MR. Current and future perspectives of liquid biopsies in genomics-driven oncology. Nat Rev Genet. (2019) 20:71–88. doi: 10.1038/s41576-018-0071-5 30410101

[68] Wood DE White JR Georgiadis A Van Emburgh B Parpart-Li S Mitchell J. A machine learning approach for somatic mutation discovery. Sci Transl Med. (2018) 10:eaar7939. doi: 10.1126/scitranslmed.aar7939 30185652 PMC6481619

[69] Louis DN Perry A Reifenberger G Von Deimling A Figarella-Branger D Cavenee WK. The 2016 world health organization classification of tumors of the central nervous system: a summary. Acta Neuropathol. (2016) 131:803–20. doi: 10.1007/s00401-016-1545-1 27157931

[70] Liu S Shah Z Sav A Russo C Berkovsky S Qian Y. Isocitrate dehydrogenase (IDH) status prediction in histopathology images of gliomas using deep learning. Sci Rep. (2020) 10(1):7733. doi: 10.1038/s41598-020-64588-y 32382048 PMC7206037

[71] Bai H Wang Y Liu H Lu J. Development of a four-mRNA expression-based prognostic signature for cutaneous melanoma. Front Genet. (2021) 12:680617. doi: 10.3389/fgene.2021.680617 34335689 PMC8320537

[72] Decalf J Albert ML Ziai J. New tools for pathology: a user’s review of a highly multiplexed method for in *situ* analysis of protein and RNA expression in tissue. J Pathol. (2019) 247:650–61. doi: 10.1002/path.5223 30570141

[73] Du Z Lin JR Rashid R Maliga Z Wang S Aster JC. Qualifying antibodies for image-based immune profiling and multiplexed tissue imaging. Nat Protoc. (2019) 14(10):2900–30. doi: 10.1038/s41596-019-0206-y PMC6959005 31534232

[74] Huss R Coupland SE. Software-assisted decision support in digital histopathology. J Pathol. (2020) 250:685–92. doi: 10.1002/path.5388 31994192

[75] Colling R Pitman H Oien K Rajpoot N Macklin P CM-Path AI in Histopathology Working Group. Artificial intelligence in digital pathology: a roadmap to routine use in clinical practice. J Pathol. (2019) 249(2):143–50. doi: 10.1002/path.5310 31144302

[76] Wickenhauser C Bethmann D Feng Z Jensen SM Ballesteros-Merino C Massa C. Multispectral fluorescence imaging allows for distinctive topographic assessment and subclassification of tumor-infiltrating and surrounding immune cells. Methods Protoc. (2019) 1913:13–31. doi: 10.1007/978-1-4939-8979-9_2 30666596

[77] Chen GM Azzam A Ding Y-Y Barrett DM Grupp SA Tan K. Dissecting the tumor–immune landscape in chimeric antigen receptor T-cell therapy: key challenges and opportunities for a systems immunology approach. Clin Cancer Res. (2020) 26:3505–13. doi: 10.1158/1078-0432.CCR-19-3888 PMC7367708 32127393

[78] Handy CE Antonarakis ES. Sipuleucel-T for the treatment of prostate cancer: novel insights and future directions. Future Oncol. (2018) 14:907–17. doi: 10.2217/fon-2017-0531 PMC5925432 29260582

[79] Yang Y Zhao Y Liu X Huang J. Artificial intelligence for prediction of response to cancer immunotherapy. Semin Cancer Biol. (2022) 87:137–47. doi: 10.1016/j.semcancer.2022.11.008 36372326

[80] Mu W Jiang L Zhang J Shi Y Gray JE Tunali I. Non-invasive decision support for NSCLC treatment using PET/CT radiomics. Nat Commun. (2020) 11(1):5228. doi: 10.1038/s41467-020-19116-x 33067442 PMC7567795

[81] Poplin R Chang PC Alexander D Schwartz S Colthurst T Ku A. A universal SNP and small-indel variant caller using deep neural networks. Nat Biotechnol. (2018) 36(10):983–7. doi: 10.1038/nbt.4235 30247488

[82] Chiu R Nip KM Birol I. Fusion-Bloom: fusion detection in assembled transcriptomes. Bioinformatics. (2020) 36:2256–7. doi: 10.1093/bioinformatics/btz902 PMC7141844 31790154

[83] Diao K Chen J Wu T Wang X Wang G Sun X. Seq2Neo: A comprehensive pipeline for cancer neoantigen immunogenicity prediction. Int J Mol Sci. (2022) 23(19):11624. doi: 10.3390/ijms231911624 36232923 PMC9569519

[84] Wang G Wan H Jian X Li Y Ouyang J Tan X. INeo-epp: A novel T-cell HLA class-I immunogenicity or neoantigenic epitope prediction method based on sequence-related amino acid features. BioMed Res Int. (2020) 2020:1–12. doi: 10.1155/2020/5798356 PMC7315274 32626747

[85] Science - 3T Biosciences - TCR Target ID and Therapeutic Discovery.” 3T Biosciences - TCR Target ID And Therapeutic Discovery. Available online at: https://3tbiosciences.com/ (Accessed October 13, 2023).

[86] Mees W. “Home.” myNEO Therapeutics (2023). Available online at: https://myneotx.com/.

[87] Pounraj S Chen S Ma L Mazzieri R Dolcetti R Rehm BHA. Targeting tumor heterogeneity with neoantigen-based cancer vaccines. Cancer Res. (2023) 84:353–363. doi: 10.1158/0008-5472.CAN-23-2042 38055891

[88] Morinaga T Inozume T Kawazu M Ueda Y Sax N Yamashita K. Mixed response to cancer immunotherapy is driven by intratumor heterogeneity and differential interlesion immune infiltration. Cancer Res Commun. (2022) 2(7):739–53. doi: 10.1158/2767-9764.CRC-22-0050 PMC10010332 36923281

[89] Dimitrov I Bangov I Flower DR Doytchinova I. AllerTOP v.2—a server for in silico prediction of allergens. J Mol Model. (2014) 20:2278. doi: 10.1007/s00894-014-2278-5 24878803

[90] Gasteiger E. Protein identification and analysis tools on the exPASy server’. In: Totowa NJ, editor. The Proteomics Protocols Handbook. Humana Press (2005). p. 571–607. doi: 10.1385/1-59259-890-0:571

[91] Kaushik R Kant R Christodoulides M. Artificial intelligence in accelerating vaccine development - current and future perspectives. Front Bacteriology. (2023) 2:1258159. doi: 10.3389/fbrio.2023.1258159

[92] Magnan CN Zeller M Kayala MA Vigil A Randall A Felgner PL. High-throughput prediction of protein antigenicity using protein microarray data. Bioinformatics. (2010) 26(23):2936–43. doi: 10.1093/bioinformatics/btq551 PMC2982151 20934990

[93] Doytchinova IA Flower DR. VaxiJen: a server for prediction of protective antigens, tumour antigens and subunit vaccines. BMC Bioinf. (2007) 8:4. doi: 10.1186/1471-2105-8-4 PMC1780059 17207271

[94] Hu Z Ott PA Wu CJ. Towards personalized, tumour-specific, therapeutic vaccines for cancer. Nat Rev Immunol. (2018) 18:168–82. doi: 10.1038/nri.2017.131 PMC6508552 29226910

[95] Bowen WS Svrivastava AK Batra L Barsoumian H Shirwan H. Current challenges for cancer vaccine adjuvant development. Expert Rev Vaccines. (2018) 17:207–15. doi: 10.1080/14760584.2018.1434000 PMC6093214 29372660

[96] Tiriveedhi V Tucker N Herndon J Li L Sturmoski M Ellis M. Safety and preliminary evidence of biologic efficacy of a mammaglobin-A DNA vaccine in patients with stable metastatic breast cancer. Clin Cancer Res. (2014) 20(23):5964–75. doi: 10.1158/1078-0432.CCR-14-0059 PMC4322416 25451106

[97] Trimble CL Morrow MP Kraynyak KA Shen X Dallas M Yan J. Safety, efficacy, and immunogenicity of VGX-3100, a therapeutic synthetic DNA vaccine targeting human papillomavirus 16 and 18 E6 and E7 proteins for cervical intraepithelial neoplasia 2/3: a randomised, double-blind, placebo-controlled phase 2b trial. Lancet. (2015) 386:2078–88. doi: 10.1016/S0140-6736(15)00239-1 PMC4888059 26386540

[98] Kutzler MA Weiner DB. DNA vaccines: ready for prime time? Nat Rev Genet. (2008) 9:776–88. doi: 10.1038/nrg2432 PMC4317294 18781156

[99] Sack BK Herzog RW. Evading the immune response upon in *vivo* gene therapy with viral vectors. Curr Opin Mol Ther. (2009) 11:493–503. 19806497 PMC3584155

[100] Lopes A Vandermeulen G Préat V. Cancer DNA vaccines: current preclinical and clinical developments and future perspectives. J Exp Clin Cancer Res. (2019) 38:146. doi: 10.1186/s13046-019-1154-7 30953535 PMC6449928

[101] Long GV Ferrucci PF Khattak A Meniawy TM Ott PA Chisamore M. KEYNOTE – D36: personalized immunotherapy with a neoepitope vaccine, EVX-01 and pembrolizumab in advanced melanoma. Future Oncol. (2022) 18(31):3473–80. doi: 10.2217/fon-2022-0694 36047545

[102] Evaxion biotech . Available online at: https://www.evaxion-biotech.com/ (Accessed Sep. 13, 2024).

[103] Keshavarzi Arshadi A Salem M. Artificial intelligence in medicine. AI Immunoinformatics. (2020), 1–9. doi: 10.1007/978-3-030-58080-3_113-1

[104] Iurescia S Fioretti D Fazio VM Rinaldi M. Epitope-driven DNA vaccine design employing immunoinformatics against B-cell lymphoma: A biotech’s challenge. Biotechnol Adv. (2012) 30:372–83. doi: 10.1016/j.bioteChadv.2011.06.020 21745560

[105] Akbari P Gavidel P Gardaneh M. The revolutionizing impact of artificial intelligence on breast cancer management. Arch Breast Cancer. (2019) 6:1–3. doi: 10.32768/abc.201961-3

[106] Bianchini F. The problem of prediction in artificial intelligence and synthetic biology. Complex Syst. (2018) 27:249–66. doi: 10.25088/ComplexSystems.27.3.249

[107] Allemailem KS Alsahli MA Almatroudi A Alrumaihi F Al Abdulmonem W Moawad AA. Innovative strategies of reprogramming immune system cells by targeting CRISPR/Cas9-based genome-editing tools: A new era of cancer management. Int J Nanomedicine. (2023) 18:5531–59. doi: 10.2147/IJN.S424872 PMC10547015 37795042

[108] Singh SP Mishra BN. Prediction of MHC binding peptide using Gibbs motif sampler, weight matrix and artificial neural network. Bioinformation. (2008) 3:150–5. doi: 10.6026/97320630003150 PMC2639663 19238237

[109] Conforti A Salvatori E Lione L Compagnone M Pinto E Shorrock C. Linear DNA amplicons as a novel cancer vaccine strategy. J Exp Clin Cancer Res. (2022) 41(1):195. doi: 10.1186/s13046-022-02402-5 35668533 PMC9169303

[110] Costa K. Codon optimization and AI: Tackling a classic synthetic biology problem . Available online at: https://www.absci.com/ai-codon-optimization-synthetic-biology/ (Accessed Sep. 13, 2024).

[111] Xu S Yang K Li R Zhang L. mRNA vaccine era—Mechanisms, drug platform and clinical prospection. Int J Mol Sci. (2020) 21:6582. doi: 10.3390/ijms21186582 32916818 PMC7554980

[112] Vormehr M Türeci Ö Sahin U. Harnessing tumor mutations for truly individualized cancer vaccines. Annu Rev Med. (2019) 70:395–407. doi: 10.1146/annurev-med-042617-101816 30691374

[113] Antonarelli G Corti C Tarantino P Ascione L Cortes J Romero P. Therapeutic cancer vaccines revamping: technology advancements and pitfalls. Ann Oncol. (2021) 32(12):1537–51. doi: 10.1016/j.annonc.2021.08.2153 PMC8420263 34500046

[114] Zhan X Wang B Wang Y Chen L Peng X Li J. Phase I trial of personalized mRNA vaccine encoding neoantigen in patients with advanced digestive system neoplasms. J Clin Oncol. (2020) 38:e15269–9. doi: 10.1200/JCO.2020.38.15_suppl.e15269

[115] Mekki-Berrada F Ren Z Huang T Wong WK Zheng F Xie J. Two-step machine learning enables optimized nanoparticle synthesis. NPJ Comput Mater. (2021) 7(1):55. doi: 10.1038/s41524-021-00520-w

[116] Fu H Liang Y Zhong X Pan Z Huang L Zhang H. Codon optimization with deep learning to enhance protein expression. Sci Rep. (2020) 10(1):17617. doi: 10.1038/s41598-020-74091-z 33077783 PMC7572362

[117] Pappalardo F Russo G Reche PA. Toward computational modelling on immune system function. BMC Bioinf. (2020) 21:546. doi: 10.1186/s12859-020-03897-5 PMC7733695 33308137

[118] Singh J Hanson J Paliwal K Zhou Y. RNA secondary structure prediction using an ensemble of two-dimensional deep neural networks and transfer learning. Nat Commun. (2019) 10:5407. doi: 10.1038/s41467-019-13395-9 31776342 PMC6881452

[119] Yu H Qi Y Ding Y. Deep learning in RNA structure studies. Front Mol Biosci. (2022) 9:869601. doi: 10.3389/fmolb.2022.869601 35677883 PMC9168262

[120] He S Gao B Sabnis R Sun Q. RNAdegformer: accurate prediction of mRNA degradation at nucleotide resolution with deep learning. Brief Bioinform. (2023) 24:bbac581. doi: 10.1093/bib/bbac581 36633966 PMC9851316

[121] Costa V Angelini C De Feis I Ciccodicola A. Uncovering the complexity of transcriptomes with RNA-seq. J BioMed Biotechnol. (2010) 2010:1–19. doi: 10.1155/2010/853916 PMC2896904 20625424

[122] HIPC-CHI Signatures Project Team, HIPC-I Consortium. Multicohort analysis reveals baseline transcriptional predictors of influenza vaccination responses. Sci Immunol. (2017) 2(14):eaal4656. doi: 10.1126/sciimmunol.aal4656 28842433 PMC5800877

[123] Dart A. Organoid diversity. Nat Rev Cancer. (2018) 18:404–5. doi: 10.1038/s41568-018-0018-3 29700397

[124] Xie J Tian W Tang Y Zou Y Zheng S Wu L. Establishment of a cell necroptosis index to predict prognosis and drug sensitivity for patients with triple-negative breast cancer. Front Mol Biosci. (2022) 9:834593. doi: 10.3389/fmolb.2022.834593 35601830 PMC9117653

[125] Huemer F Leisch M Geisberger R Melchardt T Rinnerthaler G Zaborsky N. Combination strategies for immune-checkpoint blockade and response prediction by artificial intelligence. Int J Mol Sci. (2020) 21(8):2856. doi: 10.3390/ijms21082856 32325898 PMC7215892

[126] Mizukami T Momose H Kuramitsu M Takizawa K Araki K Furuhata K. System vaccinology for the evaluation of influenza vaccine safety by multiplex gene detection of novel biomarkers in a preclinical study and batch release test. PloS One. (2014) 9(7):e101835. doi: 10.1371/journal.pone.0101835 25010690 PMC4092028

[127] de Jongh RPH van Dijk ADJ Julsing MK Schaap PJ Ridder D. Designing eukaryotic gene expression regulation using machine learning. Trends Biotechnol. (2020) 2:191–201. doi: 10.1016/j.tibtech.2019.07.007 31431299

[128] Svensson V. Droplet scRNA-seq is not zero-inflated. Nat Biotechnol. (2020) 38:147–50. doi: 10.1038/s41587-019-0379-5 31937974

[129] Zeng H Edwards MD Liu G Gifford DK. Convolutional neural network architectures for predicting DNA–protein binding. Bioinformatics. (2016) 32:i121–7. doi: 10.1093/bioinformatics/btw255 PMC4908339 27307608

[130] Avsec Ž Agarwal V Visentin D Ledsam JR Grabska-Barwinska A Taylor KR. Effective gene expression prediction from sequence by integrating long-range interactions. Nat Methods. (2021) 18(10):1196–203. doi: 10.1038/s41592-021-01252-x PMC8490152 34608324

[131] Ji Y Zhou Z Liu H Davuluri RV. DNABERT: pre-trained Bidirectional Encoder Representations from Transformers model for DNA-language in genome. Bioinformatics. (2021) 37:2112–20. doi: 10.1093/bioinformatics/btab083 PMC11025658 33538820

[132] Moderna . Available online at: https://www.modernatx.com/research/product-pipeline (Accessed : Sep. 13, 2024).

[133] Ramón y Cajal S Sesé M Capdevila C Aasen T De Mattos-Arruda L Diaz-Cano SJ. Clinical implications of intratumor heterogeneity: challenges and opportunities. J Mol Med. (2020) 98:161–77. doi: 10.1007/s00109-020-01874-2 PMC7007907 31970428

[134] Dunn GP Old LJ Schreiber RD. The immunobiology of cancer immunosurveillance and immunoediting. Immunity. (2004) 21:137–48. doi: 10.1016/j.immuni.2004.07.017 15308095

[135] Miranda A Hamilton PT Zhang AW Pattnaik S Becht E Mezheyeuski A. Cancer stemness, intratumoral heterogeneity, and immune response across cancers. Proc Natl Acad Sci. (2019) 116(18):9020–9. doi: 10.1073/pnas.1818210116 PMC6500180 30996127

[136] Stephens AJ Burgess-Brown NA Jiang S. Beyond just peptide antigens: the complex world of peptide-based cancer vaccines. Front Immunol. (2021) 12:696791. doi: 10.3389/fimmu.2021.696791 34276688 PMC8279810

[137] Mohanty E Mohanty A. Role of artificial intelligence in peptide vaccine design against RNA viruses. Inform Med Unlocked. (2021) 26:100768. doi: 10.1016/j.imu.2021.100768 34722851 PMC8536498

[138] Chen Z Zhang S Han N Jiang J Xu Y Ma D. A neoantigen-based peptide vaccine for patients with advanced pancreatic cancer refractory to standard treatment. Front Immunol. (2021) 12:691605. doi: 10.3389/fimmu.2021.691605 34484187 PMC8414362

[139] Moldovan I Targoni O Zhang W Sundararaman S Lehmann PV. How frequently are predicted peptides actually recognized by CD8 cells? Cancer Immunology Immunotherapy. (2016) 65:847–55. doi: 10.1007/s00262-016-1840-7 PMC4917593 27108305

[140] Desrichard A Snyder A Chan TA. Cancer neoantigens and applications for immunotherapy. Clin Cancer Res. (2016) 22:807–12. doi: 10.1158/1078-0432.CCR-14-3175 26515495

[141] Skwarczynski M Toth I. Peptide-based synthetic vaccines. Chem Sci. (2016) 7:842–54. doi: 10.1039/C5SC03892H PMC5529997 28791117

[142] Janeway CA Jr Travers P Walport M. The Immune System in Health and Disease, 5th ed. New York: The Immune System in Health and Disease (2001).

[143] Malonis RJ Lai JR Vergnolle O. Peptide-based vaccines: current progress and future challenges. Chem Rev. (2020) 120:3210–29. doi: 10.1021/acs.chemrev.9b00472 PMC7094793 31804810

[144] Reed SG Orr MT Fox CB. Key roles of adjuvants in modern vaccines. Nat Med. (2013) 19:1597–608. doi: 10.1038/nm.3409 24309663

[145] Tomic A Tomic I Rosenberg-Hasson Y Dekker CL Maecker HT Davis MM. SIMON, an automated machine learning system, reveals immune signatures of influenza vaccine responses. J Immunol. (2019) 203:749–59. doi: 10.4049/jimmunol.1900033 PMC6643048 31201239

[146] Li F Han J Cao T Lam W Fan B Tang W. Design of self-assembly dipeptide hydrogels and machine learning *via* their chemical features. Proc Natl Acad Sci. (2019) 116(23):11259–64. doi: 10.1073/pnas.1903376116 PMC6561259 31110004

[147] Fujiwara Y Okada K Omori T Sugimura K Miyata H Ohue M. Multiple therapeutic peptide vaccines for patients with advanced gastric cancer. Int J Oncol. (2017) 50(5):1655–62. doi: 10.3892/ijo.2017.3955 28393243

[148] Ardigen.com . Available online at: https://ardigen.com/wp-content/uploads/2023/01/Immunology-2023_-Design-peptide-based-vaccines.pdf (Accessed Sep. 14, 2024).

[149] Bujak J Kłęk S Balawejder M Kociniak A Wilkus K Szatanek R. Creating an innovative artificial intelligence-based technology (TCRact) for designing and optimizing T cell receptors for use in cancer immunotherapies: protocol for an observational trial. JMIR Res Protoc. (2023) 12(1):e45872. doi: 10.2196/45872 37440307 PMC10375398

[150] Sanecka-Duin Biernat P Czarnocka W Dudaniec K Gniewek O Grochowalski Ł. AI-based tools for target identification foster the generation of novel TCR hits against solid tumor antigens.

[151] Santos PM Butterfield LH. Dendritic cell–based cancer vaccines. J Immunol. (2018) 200:443–9. doi: 10.4049/jimmunol.1701024 PMC5880540 29311386

[152] Vik-Mo EO Nyakas M Mikkelsen BV Moe MC Due-Tønnesen P Suso EM. Therapeutic vaccination against autologous cancer stem cells with mRNA-transfected dendritic cells in patients with glioblastoma. Cancer Immunology Immunotherapy. (2013) 62:1499–509. doi: 10.1007/s00262-013-1453-3 PMC3755221 23817721

[153] Subtirelu RC Teichner EM Ashok A Parikh C Talasila S Matache IM. Advancements in dendritic cell vaccination: enhancing efficacy and optimizing combinatorial strategies for the treatment of glioblastoma. Front Neurol. (2023) 14:1271822. doi: 10.3389/fneur.2023.1271822 38020665 PMC10644823

[154] Lin MJ Svensson-Arvelund J Lubitz GS Marabelle A Melero I Brown BD. Cancer vaccines: the next immunotherapy frontier. Nat Cancer. (2022) 3(8):911–26. doi: 10.1038/s43018-022-00418-6 35999309

[155] Mirsanei Z Habibi S Kheshtchin N Mirzaei R Arab S Zand B. Optimized dose of dendritic cell-based vaccination in experimental model of tumor using artificial neural network. Iran J Allergy Asthma Immunol. (2020), 172–82. doi: 10.18502/ijaai.v19i2.2770 32372630

[156] Gao Y Wang Z Cui Y Xu M Weng L. Emerging strategies of engineering and tracking dendritic cells for cancer immunotherapy. ACS Appl Bio Mater. (2023) 6:24–43. doi: 10.1021/acsabm.2c00790 36520013

[157] Paulis LE Mandal S Kreutz M Figdor CG. Dendritic cell-based nanovaccines for cancer immunotherapy. Curr Opin Immunol. (2013) 25:389–95. doi: 10.1016/j.coi.2013.03.001 23571027

[158] Hashemi V Farhadi S Chaleshtari MG Seashore-Ludlow B Masjedi A Hojjat-Farsangi M. Nanomedicine for improvement of dendritic cell-based cancer immunotherapy. Int Immunopharmacol. (2020) 83:106446. doi: 10.1016/j.intimp.2020.106446 32244048

[159] Thakur N Thakur S Chatterjee S Das J Sil PC. Nanoparticles as smart carriers for enhanced cancer immunotherapy. Front Chem. (2020) 8:597806. doi: 10.3389/fchem.2020.597806 33409265 PMC7779678

[160] Baghaei B Saeb MR Jafari SH Khonakdar HA Rezaee B Goodarzi V. Modeling and closed-loop control of particle size and initial burst of PLGA biodegradable nanoparticles for targeted drug delivery. J Appl Polym Sci. (2017) 134(33):45145. doi: 10.1002/app.45145

[161] Lin Z Chou W-C Cheng Y-H He C Monteiro-Riviere NA Riviere JE. Predicting nanoparticle delivery to tumors using machine learning and artificial intelligence approaches. Int J Nanomedicine. (2022) 17:1365–79. doi: 10.2147/IJN.S344208 PMC8961007 35360005

[162] Yingling YG Shapiro BA. Computational design of an RNA hexagonal nanoring and an RNA nanotube. Nano Lett. (2007) 7:2328–34. doi: 10.1021/nl070984r 17616164

[163] Moore JA Chow JCL. Recent progress and applications of gold nanotechnology in medical biophysics using artificial intelligence and mathematical modeling. Nano Express. (2021) 2:022001. doi: 10.1088/2632-959X/abddd3

[164] Suberi AAM Zakaria WNW Tomari R. Dendritic cell recognition in computer aided system for cancer immunotherapy. Proc Comput Sci. (2017) 105:177–82. doi: 10.1016/j.procs.2017.01.201

[165] Weber JS Matteo SC Adnan K Tarek M George A Matthew H. “Individualised neoantigen therapy mRNA-4157 (V940) plus pembrolizumab versus pembrolizumab monotherapy in resected melanoma (KEYNOTE-942): a randomised, phase 2b study.” The Lancet (2024) 403:632–44. 10.1016/S0140-6736(23)02268-7 38246194

[166] A Study of mRNA-5671/V941 as Monotherapy and in Combination with Pembrolizumab (V941-001) . Available online at: https://classic.clinicaltrials.gov/ct2/show/NCT03948763 (Accessed Sep. 14, 2024).

[167] Powderly JD Sullivan RJ Gutierrez M Khattak A Thomas SS Jimeno A. Phase 1/2 study of mRNA-4359 administered alone and in combination with immune checkpoint blockade in adult participants with advanced solid tumors. J Clin Oncol. (2023) 41:TPS2676–TPS2676. doi: 10.1200/JCO.2023.41.16_suppl.TPS2676

[168] Hillemanns P Baurain JF Blecharz P Lindemann K Nicolaisen B Schetne K. 881TiP A multi-centre, open-label phase II trial of the combination of VB10.16 and atezolizumab in patients with advanced or recurrent, non-resectable HPV16 positive cervical cancer. Ann Oncol. (2020) 31:S645–6. doi: 10.1016/j.annonc.2020.08.1020

[169] Bent A Raghavan S Dasari A Kopetz S. The future of ctDNA-defined minimal residual disease: personalizing adjuvant therapy in colorectal cancer. Clin Colorectal Cancer. (2022) 21:89–95. doi: 10.1016/j.clcc.2022.03.004 35450837 PMC9149115

[170] Delord J-P Block MS Ottensmeier C Colon-Otero G Le Tourneau C Lalanne A. Phase 1 studies of personalized neoantigen vaccine TG4050 in ovarian carcinoma (OC) and head and neck squamous cell carcinoma (HNSCC). J Clin Oncol. (2022) 40:2637–7. doi: 10.1200/JCO.2022.40.16_suppl.2637

[171] Rzhetsky A Iossifov I Koike T Krauthammer M Kra P Morris M. GeneWays: a system for extracting, analyzing, visualizing, and integrating molecular pathway data. J BioMed Inform. (2004) 37(1):43–53. doi: 10.1016/j.jbi.2003.10.001 15016385

[172] Xu Y. Deep neural networks for QSAR. Artificial intelligence in drug design (2022) 2390:233–60. doi: 10.1007/978-1-0716-1787-8_10 34731472

[173] Mayr A Klambauer G Unterthiner T Hochreiter S. DeepTox: toxicity prediction using deep learning. Front Environ Sci. (2016) 3:80. doi: 10.3389/fenvs.2015.00080

[174] Facciolà A Visalli G Laganà A Di Pietro A. An overview of vaccine adjuvants: current evidence and future perspectives. Vaccines (Basel). (2022) 10:819. doi: 10.3390/vaccines10050819 35632575 PMC9147349

[175] HogenEsch H. Mechanism of immunopotentiation and safety of aluminum adjuvants. Front Immunol. (2013) 3:406. doi: 10.3389/fimmu.2012.00406 23335921 PMC3541479

[176] Adjuvants and Vaccines | Vaccine Safety . Available online at: https://www.cdc.gov/vaccinesafety/concerns/adjuvants.html (Accessed Sep. 14, 2024).

[177] Chaudhury S Duncan EH Atre T Storme CK Beck K Kaba SA. Identification of immune signatures of novel adjuvant formulations using machine learning. Sci Rep. (2018) 8(1):17508. doi: 10.1038/s41598-018-35452-x 30504893 PMC6269591

[178] Chaudhury S Duncan EH Atre T Dutta S Spring MD Leitner WW. Combining immunoprofiling with machine learning to assess the effects of adjuvant formulation on human vaccine-induced immunity. Hum Vaccin Immunother. (2020) 16(2):400–11. doi: 10.1080/21645515.2019.1654807 PMC7062453 31589550

[179] Ma J Wang S Zhao C Yan X Ren Q Dong Z. Computer-aided discovery of potent broad-spectrum vaccine adjuvants. Angewandte Chemie Int Edition. (2023) 135(18):e202301059. doi: 10.1002/anie.202301059 36815280

[180] Ott PA Hu-Lieskovan S Chmielowski B Govindan R Naing A Bhardwaj N. A phase ib trial of personalized neoantigen therapy plus anti-PD-1 in patients with advanced melanoma, non-small cell lung cancer, or bladder cancer. Cell. (2020) 183(2):347–62.e24. doi: 10.1016/j.cell.2020.08.053 33064988

[181] Hilf N Kuttruff-Coqui S Frenzel K Bukur V Stevanović S Gouttefangeas C. Actively personalized vaccination trial for newly diagnosed glioblastoma. Nature. (2019) 565:240–5. doi: 10.1038/s41586-018-0810-y 30568303

[182] Schumacher TN Schreiber RD. Neoantigens in cancer immunotherapy. Sci (1979). (2015) 348:69–74. doi: 10.1126/science.aaa4971 25838375

[183] Coulie PG Van den Eynde BJ van der Bruggen P Boon T. Tumour antigens recognized by T lymphocytes: at the core of cancer immunotherapy. Nat Rev Cancer. (2014) 14:135–46. doi: 10.1038/nrc3670 24457417

[184] Ott PA Hu Z Keskin DB Shukla SA Sun J Bozym DJ. An immunogenic personal neoantigen vaccine for patients with melanoma. Nature. (2017) 547:217–21. doi: 10.1038/nature22991 PMC5577644 28678778

[185] Keskin DB Anandappa AJ Sun J Tirosh I Mathewson ND Li S. Neoantigen vaccine generates intratumoral T cell responses in phase Ib glioblastoma trial. Nature. (2019) 565:234–9. doi: 10.1038/s41586-018-0792-9 PMC6546179 30568305

[186] Sahin U Derhovanessian E Miller M Kloke BP Simon P Löwer M. Personalized RNA mutanome vaccines mobilize poly-specific therapeutic immunity against cancer. Nature. (2017) 547:222–6. doi: 10.1038/nature23003 28678784

[187] Zhou W-J Qu Z Song CY Sun Y Lai AL Luo MY. NeoPeptide: an immunoinformatic database of T-cell-defined neoantigens. Database. (2019) 2019:baz128. doi: 10.1093/database/baz128 31819989 PMC6901387

[188] Smith CC Chai S Washington AR Lee SJ Landoni E Field K. Machine-learning prediction of tumor antigen immunogenicity in the selection of therapeutic epitopes. Cancer Immunol Res. (2019) 7(10):1591–604. doi: 10.1158/2326-6066.CIR-19-0155 PMC6774822 31515258

[189] Oyarzún P Ellis JJ Bodén M Kobe B. PREDIVAC: CD4+ T-cell epitope prediction for vaccine design that covers 95% of HLA class II DR protein diversity. BMC Bioinf. (2013) 14:52. doi: 10.1186/1471-2105-14-52 PMC3598884 23409948

[190] Yao B Zheng D Liang S Zhang C. Conformational B-cell epitope prediction on antigen protein structures: A review of current algorithms and comparison with common binding site prediction methods. PloS One. (2013) 8:e62249. doi: 10.1371/journal.pone.0062249 23620816 PMC3631208

[191] McGranahan N Swanton C. Clonal heterogeneity and tumor evolution: past, present, and the future. Cell. (2017) 168:613–28. doi: 10.1016/j.cell.2017.01.018 28187284

[192] McGranahan N Furness AJ Rosenthal R Ramskov S Lyngaa R Saini SK. Clonal neoantigens elicit T cell immunoreactivity and sensitivity to immune checkpoint blockade. Sci (1979). (2016) 351:1463–9. doi: 10.1126/science.aaf1490 PMC4984254 26940869

[193] De Mattos-Arruda L Vazquez M Finotello F Lepore R Porta E Hundal J. Neoantigen prediction and computational perspectives towards clinical benefit: recommendations from the ESMO Precision Medicine Working Group. Ann Oncol. (2020) 31(8):978–90. doi: 10.1016/j.annonc.2020.05.008 PMC7885309 32610166

[194] Hooshmand MN Maserat E. Application of machine learning and deep learning for cancer vaccine (rapid review). Multimed Tools Appl. (2023) 83:51211–26. doi: 10.1007/s11042-023-17589-8

[195] Xia H McMichael J Becker-Hapak M Onyeador OC Buchli R McClain E. Computational prediction of MHC anchor locations guides neoantigen identification and prioritization. Sci Immunol. (2023) 8(82):eabg2200. doi: 10.1126/sciimmunol.abg2200 37027480 PMC10450883

[196] Capietto A-H Jhunjhunwala S Pollock SB Lupardus P Wong J Hänsch L. Mutation position is an important determinant for predicting cancer neoantigens. J Exp Med. (2020) 217(4). doi: 10.1084/jem.20190179 PMC7144530 31940002

[197] Battaglia S. Neoantigen prediction from genomic and transcriptomic data. Methods Enzymol. (2020) 635:267–81. doi: 10.1016/bs.mie.2019.10.003 32122550

[198] Nicolas-Boluda A Donnadieu E. Obstacles to T cell migration in the tumor microenvironment. Comp Immunol Microbiol Infect Dis. (2019) 63:22–30. doi: 10.1016/j.cimid.2018.12.006 30961814

[199] Katsikis PD Ishii KJ Schliehe C. Challenges in developing personalized neoantigen cancer vaccines. Nat Rev Immunol. (2024) 24:213–27. doi: 10.1038/s41577-023-00937-y PMC12001822 37783860

[200] Xu Y Su G-H Ma D Xiao Y Shao Z-M Jiang Y-Z. Technological advances in cancer immunity: from immunogenomics to single-cell analysis and artificial intelligence. Signal Transduct Target Ther. (2021) 6:312. doi: 10.1038/s41392-021-00729-7 34417437 PMC8377461

[201] Chen J Zhang H Zhou L Hu Y Li M He Y. Enhancing the efficacy of tumor vaccines based on immune evasion mechanisms. Front Oncol. (2021) 10:584367. doi: 10.3389/fonc.2020.584367 33614478 PMC7886973

[202] Lee RY Wu Y Goh D Tan V Ng CW Lim JC. Application of artificial intelligence to *in vitro* tumor modeling and characterization of the tumor microenvironment. Adv Healthc Mater. (2023) 12(14):2202457. doi: 10.1002/adhm.202202457 37060240

[203] Russo G Reche P Pennisi M Pappalardo F. The combination of artificial intelligence and systems biology for intelligent vaccine design. Expert Opin Drug Discovery. (2020) 15:1267–81. doi: 10.1080/17460441.2020.1791076 32662677

[204] Gao W Wang J. Synthetic micro/nanomotors in drug delivery. Nanoscale. (2014) 6:10486–94. doi: 10.1039/C4NR03124E 25096021

[205] Sarkar S V S Makuteswaran S C M. Nanobot swarm for targeted elimination of tumor in brain. ECS Trans. (2022) 107:2803–17. doi: 10.1149/10701.2803ecst

[206] Stanton SE Disis ML. Clinical significance of tumor-infiltrating lymphocytes in breast cancer. J Immunother Cancer. (2016) 4:59. doi: 10.1186/s40425-016-0165-6 27777769 PMC5067916

[207] Shameer K Johnson KW Glicksberg BS Dudley JT Sengupta PP. The whole is greater than the sum of its parts: combining classical statistical and machine intelligence methods in medicine. Heart. (2018) 104:1228–8. doi: 10.1136/heartjnl-2018-313377 29945951

[208] Stephansen JB Olesen AN Olsen M Ambati A Leary EB Moore HE. Neural network analysis of sleep stages enables efficient diagnosis of narcolepsy. Nat Commun. (2018) 9(1):5229. doi: 10.1038/s41467-018-07229-3 30523329 PMC6283836

[209] She Y Jin Z Wu J Deng J Zhang L Su H. Development and validation of a deep learning model for non–small cell lung cancer survival. JAMA Netw Open. (2020) 3(6):e205842. doi: 10.1001/jamanetworkopen.2020.5842 32492161 PMC7272121

[210] Nam JG Park S Hwang EJ Lee JH Jin KN Lim KY. Development and Validation of Deep Learning–based Automatic Detection Algorithm for Malignant Pulmonary Nodules on Chest Radiographs. Radiology. (2019) 290(1):218–28. doi: 10.1148/radiol.2018180237 30251934

